# Lactylation of Mitochondrial Adenosine Triphosphate Synthase Subunit Alpha Regulates Vascular Remodeling and Progression of Aortic Dissection

**DOI:** 10.34133/research.0799

**Published:** 2025-08-12

**Authors:** Tao Yu, Xiaolu Li, Chao Wang, Yanyan Yang, Xiuxiu Fu, Tianxiang Li, Wentao Wang, Xiangyu Liu, Xiaoxin Jiang, Ding Wei, Jian-Xun Wang

**Affiliations:** ^1^ Institute for Translational Medicine, The Affiliated Hospital of Qingdao University, Qingdao 266021, People’s Republic of China.; ^2^ Department of Cardiac Ultrasound, The Affiliated hospital of Qingdao University, Qingdao 266000, People’s Republic of China.; ^3^ Department of Cardiovascular Surgery, The Affiliated Hospital of Qingdao University, Qingdao, China.; ^4^ Department of Immunology, School of Basic Medicine, Qingdao University, Qingdao 266071, China.; ^5^ Department of General Medicine, The Affiliated Hospital of Qingdao University, Qingdao 266000, People’s Republic of China.

## Abstract

Aortic dissection (AD) is a cardiovascular disorder with a high mortality rate. Lysine Lactylation (Kla), a novel posttranslational modification, critically regulates inflammation, tumors, and cardiovascular diseases. However, its specific role in AD pathogenesis remains unexplored. Using modification omics, we conducted a macroscopic analysis of the occurrence of extensive lactylation modification in aortic dissection and identified extensive lactylation, particularly in the adenosine triphosphatase activity pathway. Among these proteins, adenosine triphosphate (ATP) synthase F1 subunit α (ATP5F1A), a subunit in the ATP synthase complex, exhibited pronounced lactylation at the K531, catalyzed by sirtuin 3 (Sirt3). Through site-directed mutagenesis (K531R/K531E), we validated the key mechanism of lactylation activation at the K531 site of ATP5F1A and the regulatory enzymes. Functionally, K531 lactylation impairs ATP synthase activity, elevates reactive oxygen species generation, reduces ATP generation, and induces mitochondrial structural abnormalities. These effects ultimately contribute to the phenotypic transformation of human aortic vascular smooth muscle cells and enhanced synthesis and secretion of matrix metalloproteinases. In addition, we assessed the potential therapeutic effect of lactylation inhibition in aortic dissection using a mouse model and a drug based in vivo lactate alteration strategy. In conclusion, targeting the lactate–Sirt3–ATP5F1A axis represents a promising therapeutic strategy for blocking the progression of aortic dissection.

## Introduction

Aortic dissection (AD) is a life-threatening condition with high morbidity and mortality rates [1,2]. Although surgical intervention is the primary treatment, substantial mortality persists because of the condition’s clinical complexity, rapid progression, and technical challenges, the mortality rate associated with surgery remains substantial [3]. Clinically, conservative drug therapy is used as an initial intervention in the early stages of acute AD, such as in cases of internal hematoma and aortic ulcer, as well as postoperatively to prevent further deterioration or recurrence [4,5]. While the pathogenesis of AD remains poorly understood, it is commonly associated with a clinicopathological feature: medial degeneration of the aortic wall [6]. However, current strategies primarily alleviate symptoms, including pain relief and blood pressure reduction, but effective therapies for preventing AD, improving medial lesions, and halting disease progression remain lacking. The lack of therapies targeting underlying mechanisms underscores the urgent need to elucidate AD etiology and develop effective disease-modifying strategies.

Lactate, a metabolite of glycolysis, is an important source of intracellular energy and intercellular signaling molecule [7,8], which serves as a fulcrum for metabolic regulation. Lactate accelerates vascular calcification through nuclear receptor subfamily 4 group A member 1-regulated mitochondrial fission and B cell lymphoma-2 (Bcl-2)-interacting protein 3-related mitophagy [9], which is related to aortic dissection [10]. Increasing evidence supports the role of lactate in regulating AD. Lactate promotes vascular smooth muscle cell (VSMC) phenotypic transformation [11], which plays a crucial role in the development of AD. In AD, VSMCs typically undergo a phenotypic switch from a contractile to a synthetic state, which is marked by the up-regulation of contractile proteins such as α-smooth muscle actin (α-SMA) and calponin [12]. This phenotypic transformation contributes to medial layer degeneration, vessel wall instability, and increased susceptibility to rupture, all of which are key features of AD [11]. In addition, lactate increasing is often used as a poor prognostic marker after surgical repair of AD [13]. Blood lactate levels can be utilized to predict the risk of acute AD rupture, postoperative mortality, and mortality after surgical repair in patients with type A acute AD [14–16]. Nevertheless, the pathological mechanisms driving acute AD remain elusive. Since its discovery in 2019, protein lysine lactylation (Kla)—particularly histone modification—has been established to regulate the expression of inflammation-related genes [17], tumors [18,19], repair of postmyocardial infarction [20], and progression of inflammation [21–24]. However, the functional impact and mechanistic basis of lactylation in AD pathogenesis await exploration.

In this study, we first conducted a lactylation-modification omics analysis of AD. The results revealed a large number of proteins with markedly increased lactylation modification in AD. Adenosine triphosphate (ATP) synthase F1 subunit α (ATP5F1A) was remarkably up-regulated, prompting us to investigate its impact on mitochondrial oxidative stress. We further delineated the regulatory axis involving site-specific lactylation at K531 and its modulation by sirtuin 3 (Sirt3).

## Results

### Lactylation has potential correlation with AD

**Fig. 1.**
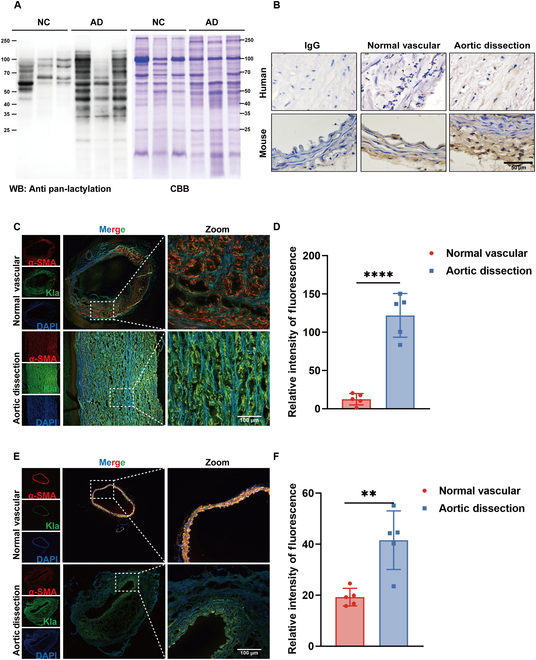
Aortic dissection is associated with lactylation. (A) The pan-lactylation immunoblots of clinically normal vascular (*n* = 3) and AD tissue (*n* = 3). CBB, Coomassie brilliant blue staining; WB, Western blot. (B) The immunohistochemistry of pan-lactylation in normal vascular and AD tissue in clinical (*n* = 3) and mouse samples (*n* = 3) (10× and 40× lens; zoom level, 2×). Scale bar, 50 μm. (C and E) The immunofluorescence costaining for α-SMA (red) with Kla (green) in normal vascular (*n* = 5) and AD tissue (*n* = 5) in clinical (C) and mouse (E) samples. Scale bars, 100 μm. (D and F) Quantification of immunofluorescence (C and E). Data are means ± SEM from 5 independent experiments and were analyzed by Student’s *t* test (D and F). **P < 0.01 and ****P < 0.0001.

Lactate affects cellular function and disease progression through lactylation modification [25]. Given that lactate levels are abnormally elevated in aortic dissection (AD), yet the specific mechanism by which it affects AD progression remains unclear, we hypothesized that lactate may promote AD through lactylation. To investigate this, we first analyzed lactylation in both clinically normal and pathological arterial tissues using Western blotting and immunohistochemistry with pan-lactylation antibodies. Our findings revealed an increased lactylation level of proteins in the dissected arteries (Fig. 1A and B). Interestingly, lactylation of cytoplasmic proteins was more pronounced than that of histones, as indicated by the Western blotting analysis showing lactylation occurring primarily in proteins with molecular weights between 20 and 200 kDa (Fig. 1A). Consistent with the results obtained in clinical tissues, the level of protein pan-lactylation in the mouse model of AD increased (Fig. 1B). Human aortic VSMCs (HAVSMCs) play a crucial role in the progression of AD, and, interestingly, extensive pan-lactylation was observed in the tunica media of blood vessels, which are primarily composed of HAVSMCs (Fig. 1C to F). These findings suggest that protein lactylation in HAVSMCs may be closely associated with the progression of AD.

### Lactylation mediated by lactate regulates the function of HAVSMCs

**Fig. 2.**
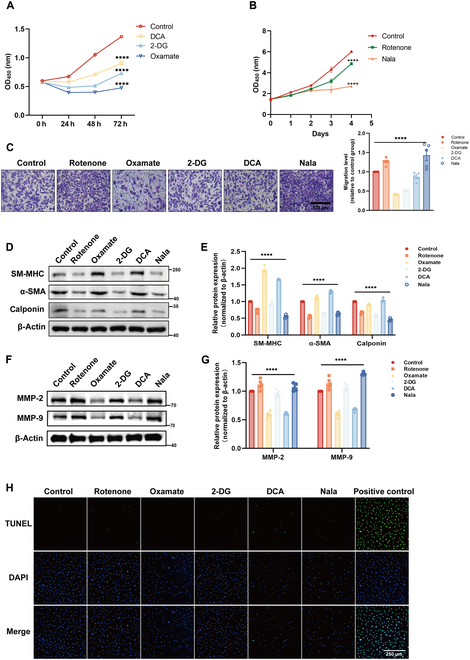
Lactylation regulates VSMC proliferation, migration, and phenotypic switching. (A) CCK-8 was performed to measure cell proliferation of VSMC after treatment with DCA (20 mM), 2-DG (10 mM), and oxamate (20 mM). OD_450_, optical density at 450 nm. (B) CCK-8 was performed to measure cell proliferation of VSMC after treatment with rotenone (10 nM) and Nala (25 mM). (C) Cell migration of VSMC under hypoxic conditions following the use of rotenone (10 nM), oxamate (20 mM), DCA (20 mM), and 2-DG (10 mM) was detected using a Transwell assay at 24 h. Scale bar, 500 μm. (D to G) Western blotting analyses and corresponding quantification were conducted to detect the protein expression level of contractile markers (D) and MMP-2/9 (E) after the use of rotenone (10 nM), oxamate (20 mM), 2-DG (10 mM), DCA (10 mM), and Nala (25 mM) in VSMC, SM-MHC, smooth muscle myosin heavy chain. (H) Detection of apoptosis in VSMC after different drug treatments using TUNEL staining. Scale bar, 500 μm. Data are means ± SEM from 3 independent experiments and were analyzed by Kruskal–Wallis test with Dunn post hoc test (A to C, E, and G). ****P < 0.0001.

Endogenous lactate serves as the substrate for lactylation. Lactate dehydrogenase A (LDHA) and LDHB are classic enzymes involved in lactate metabolism, with LDHA catalyzing the conversion of pyruvate to lactate and LDHB mediating the reverse reaction of lactate to pyruvate [26]. To investigate the impact of these enzymes on intracellular lactate levels and lactylation in HAVSMCs, we measured intracellular lactate levels following the knockdown of LDHA. Knocking down LDHA significantly reduced intracellular lactate levels and protein lactylation (Fig. S1B). In addition, we modulated lactate levels using in vitro methods. Different concentrations of sodium dichloroacetate (DCA), 2-deoxy-d-glucose (2-DG), and oxamate were used to treat HAVSMCs for 24 h. The results showed that lactate concentration in cells decreased after treatment with these drugs, and, correspondingly, the level of protein lactylation decreased (Fig. S1C to H). Conversely, after treatment with rotenone and sodium lactate (Nala) at different concentrations, intracellular lactate and lactylation levels in HAVSMCs increased (Fig. S2). To further investigate the effects of lactate and lactylation on cellular function, we assessed cell activity following treatment with these drugs. Regardless of whether the drug increased or decreased intracellular lactate levels, cell viability was inhibited, possibly because of the cytotoxic effects of the drugs (Fig. 2A and B). Transwell assay showed that a decrease in lactylation reduced the migration of HAVSMCs and, likewise, an increase in lactylation increased their migration (Fig. 2C). Therefore, changes in lactylation affected the expression of contractile markers major histocompatibility complex (MHC), α-SMA, and calponin (Fig. 2D and E), along with the changes in expression of matrix metalloproteinase 2 (MMP-2) and MMP-9 (Fig. 2F and G) in HAVSMCs. These results indicate that an increase in lactylation (cells treatment with rotenone and Nala) can reduce contractile phenotypic of HAVSMCs but promote MMP-2 and MMP-9 production. In addition, alterations in lactate levels had a modest effect on HAVSMC apoptosis (Fig. 2H).

### Inhibition of lactylation in vivo can reduce AD

**Fig. 3.**
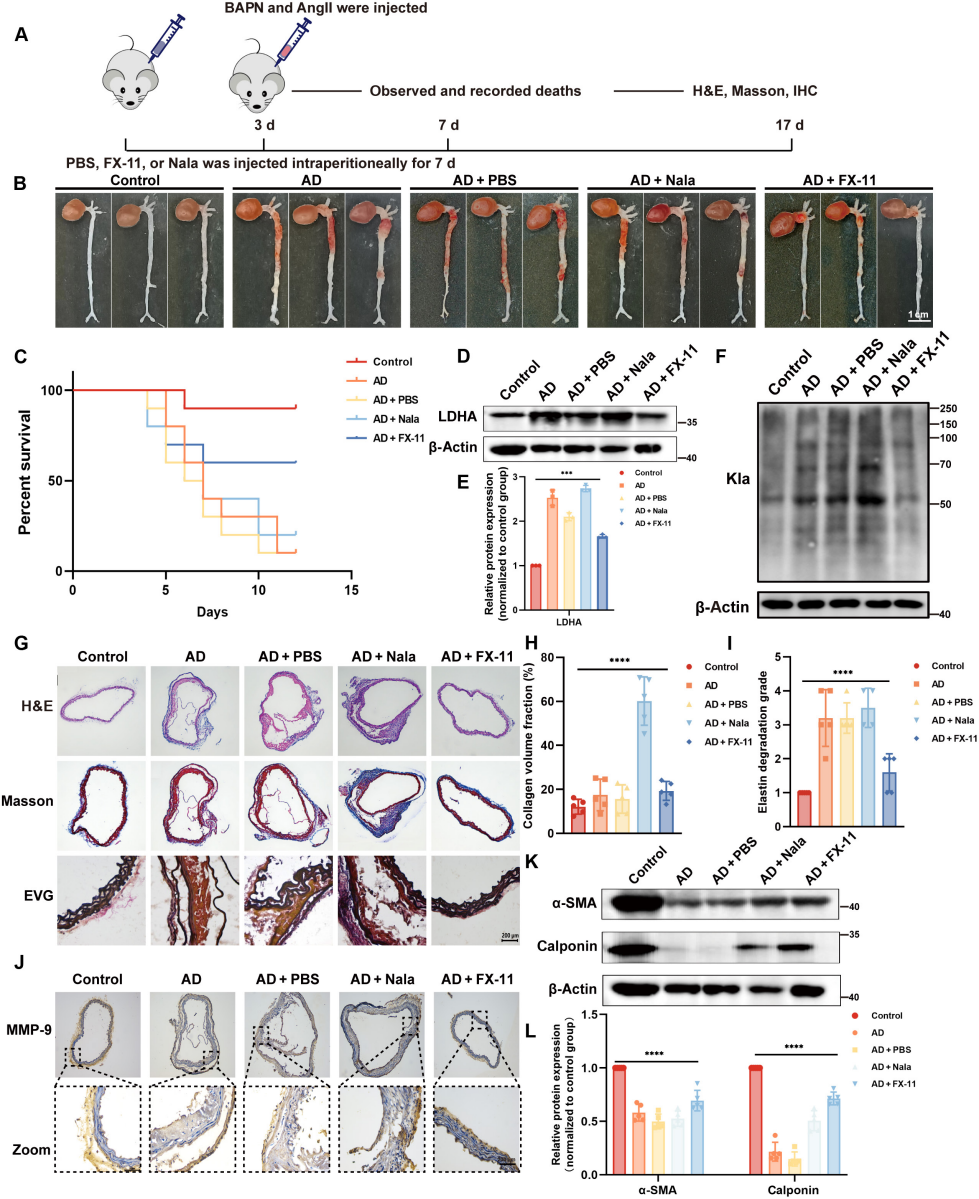
Effect of lactylation on aortic dissection in mice. (A) AD mice were constructed by injecting BAPN and AngII, and PBS, FX-11 (4 mg/kg/d), or Nala (1 g/kg/d) was injected intraperitoneally for 7 days (*n* = 11). (B) Typical images showed macroscopic features of isolated mice aorta from different groups (*n* = 3). Scale bar, 1 cm. (C) Survival rate curve of each group of mice. (D and E) Detection of LDHA expression levels in different groups. (F) Western blot was used to detect the lactylation level of the aorta of mice in different groups. (G to I) Representative images of H&E, EVG, and Masson staining in thoracic arteries obtained from mice in different groups (*n* = 5). Scale bar, 200 μm. (J) Immunohistochemical (IHC) staining of MMP-9 in different groups. (K and L) Western blot was used to detect the marker of contractile phenotype mice in different groups. Data are means ± SEM from 3 or 5 independent experiments and were analyzed by Kruskal–Wallis test with Dunn post hoc test (D, H, and K). ***P < 0.001 and ****P < 0.0001.

We examined the effect of lactylation on AD progression, according to the scheme shown in Fig. 3A. Mice received either lactate or FX-11 (an LDHA inhibitor) 3 days before the induction of acute AD. Fourteen days postintraperitoneal injection of angiotensin II (AngII) and B-aminopropionitrile (BAPN), we observed that FX-11 treatment significantly reduced both the severity of the lesion and the mortality rate in mice induced by AngII and BAPN (Fig. 3B and C). Western blotting showed that the LDHA expression was elevated in AD, and the FX-11 can inhibit this phenomenon (Fig. 3D and E). In parallel, lactylation levels were higher in the AD group, and lactate supplementation further increased lactylation, whereas LDHA inhibition with FX-11 reduced lactylation levels (Fig. 3F). Furthermore, AngII- and BAPN-treated mice exhibited more pronounced degradation of elastic fibers and interstitial fibrosis, which was alleviated by FX-11 treatment (Fig. 3G to I). The increase in lactylation in vivo inhibited the expression of contractile phenotype proteins and promoted MMP-9 levels (Fig. 3J to L). However, FX-11 treatment inhibited the phenotypic transformation and MMP-9 expression. Therefore, FX-11-mediated inhibition of lactylation can reverse the pathological process of AD, thereby reducing AD-induced mortality.

### ATP5F1A may be the key protein for lactylation in AD

**Fig. 4.**
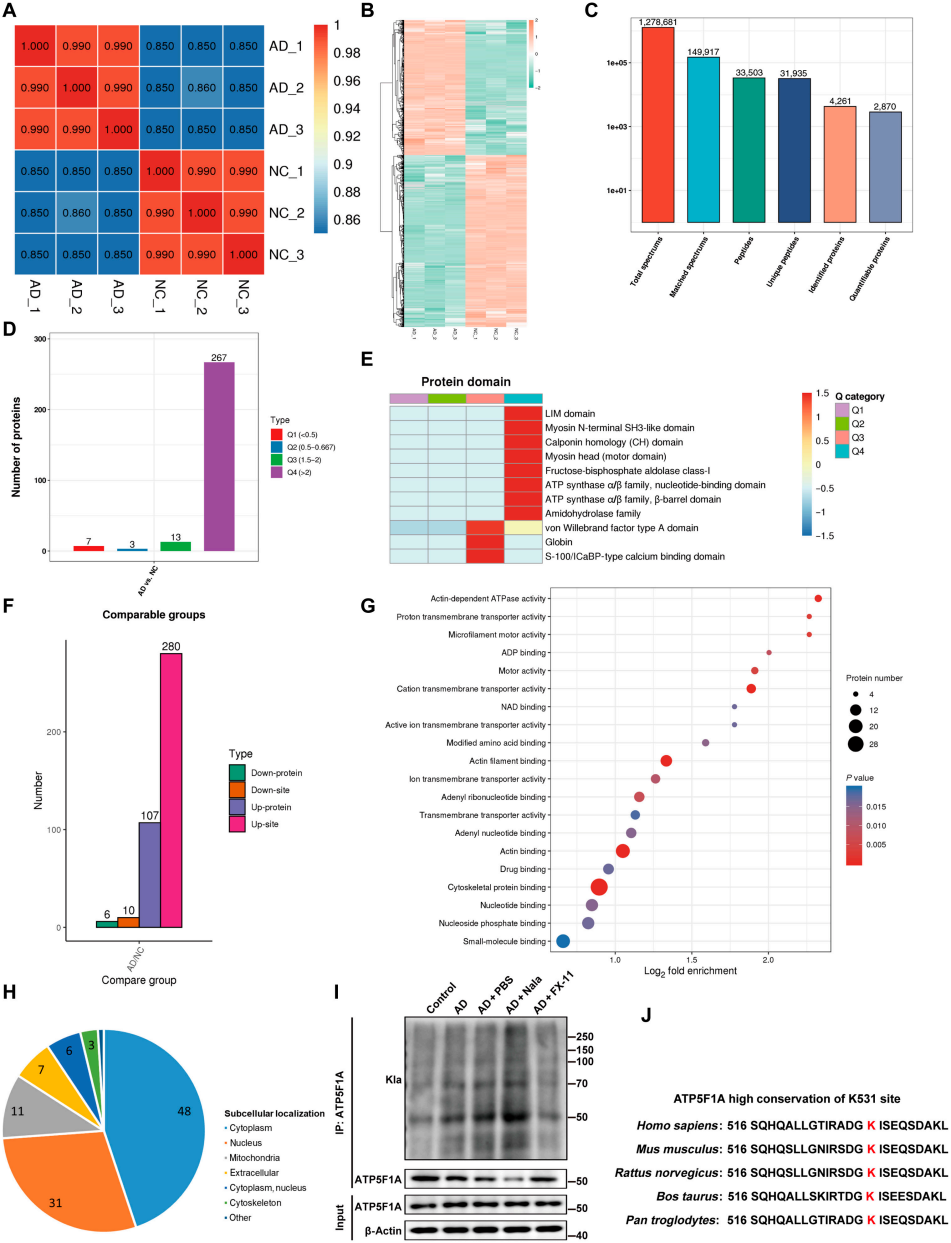
Lactylation analysis. (A) The heatmap drawn by calculating the Pearson’s correlation coefficient between all samples in pairs (*n* = 3). This coefficient is a value that measures the degree of linear correlation between 2 sets of data: the closer the Pearson coefficient is to −1, the stronger the negative correlation; the closer it is to 1, the stronger the positive correlation; and the closer it is to 0, the weaker the correlation. (B) Modification omics differential analysis heatmap. (C) Statistical map of differential proteins and sites. (D) Cluster analysis of differentially expressed proteins, dividing them into 4 parts based on their differential expression multiples, called Q1 to Q4. (E) On the basis of the *P* value of Fisher’s exact test obtained from enrichment analysis, the relevant functions in different groups were clustered together using hierarchical clustering method and plotted as a heatmap. The horizontal direction of the heatmap represents the enrichment test results of different groups, while the vertical direction represents the description of differentially expressed enrichment-related functions (protein domain). The color blocks corresponding to different groups of differentially expressed proteins and functional descriptions indicate the degree of enrichment. Red represents strong enrichment, while blue represents weak enrichment. SH3, src homology 3; ICaBP, intestinal calcium binding protein‌. (F) Numbers of Kla sites exhibiting remarkable different Kla levels and numbers of proteins exhibiting remarkable protein-level changes in normal artery and aortic dissection. (G) Gene Ontology pathway of differentially modified proteins. NAD, nicotinamide adenine dinucleotide. (H) Subcellular localization of differentially modified proteins. (I) Detection of lactylation levels of ATP5F1A in different groups using IP. (J) Conservative analysis of ATP5F1A K531 site among different species.

Next, we collected normal vessels and AD tissues, using 4-dimensional (4D) label-free proteomics to identify proteins undergoing lactylation (Fig. 4A and Fig. S3A). This modification omics experiment included 3 independent replicates per group, and the relative standard deviation for each group is depicted in Fig. S4A. The heatmap of differential analysis revealed increased lactylation in AD compared to normal blood vessels (Fig. 4A and B). The proteins identified with lactylation modifications and their specific sites are illustrated in Fig. 4C. Cluster analysis indicated that proteins exhibiting more than a 2-fold change were most abundant (Fig. 4D), with a notable association with the adenosine triphosphatase (ATPase) family (Fig. 4E). Mass spectrometry (MS) identified differences in lactylation of 113 proteins between the groups; 6 proteins exhibited reduced lactylation in AD tissues, while 107 showed increased lactylation (Fig. 4F and Fig. S3B), suggesting enhanced lactylation in AD proteins. Notably, over 34% of lactylation sites had increased abundance in AD tissues, with more than 25% showing over a 3-fold increase (Fig. S3C). Among these proteins, roughly 45% were cytoplasmic, 29% were nuclear, and 10% were mitochondrial (Fig. 4H). Enriched pathways included glycolysis, vascular smooth muscle contraction, and others, with the ATPase activity pathway showing the most significant differences (Fig. 4G and Fig. S3D). On the basis of these findings, we focused on ATP5F1A, a critical subunit of ATP synthase that catalyzes the conversion of adenosine diphosphate (ADP) to ATP using a proton gradient, providing cellular energy. The widespread high expression of ATP5F1A suggests its pivotal role in AD (Fig. S3E). The lactylation level of ATP5F1A was notably high in AD, with an AD/negative control (NC) ratio of 7.279, occurring at K531. Validation in animal tissues also showed that the lactylation level of ATP5F1A increased in AD and decreased after FX-11 treatment (Fig. 4I). ATP5F1A is highly conserved across mammals, and the K531 is evolutionarily conserved (Fig. 4J). These findings imply that increased lactylation at the K531 site of ATP5F1A might influence energy metabolism, thereby affecting AD.

### Lactylation modification of ATP5F1A regulates the function of HAVSMCs

**Fig. 5.**
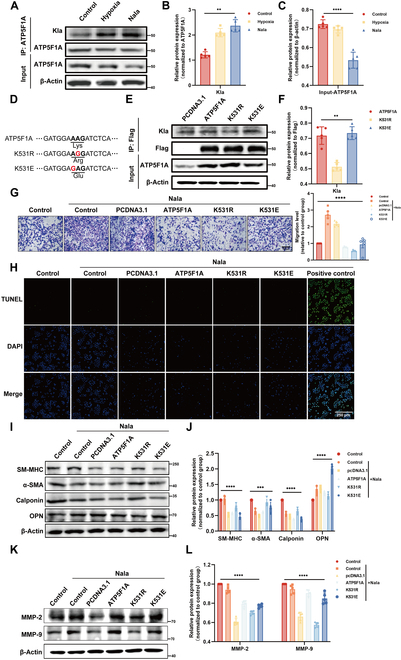
K531 lactylation of ATP5F1A regulates VSMC proliferation, migration, and phenotypic switching. (A to C) ATP5F1A expression levels were detected by Western blots after hypoxia (48 h) or Nala (25 mM) treatment. Then cell proteins were enriched by ATP5F1A antibody and detected with anti-lactyllysine antibody (*n* = 3). (D) Mutations at the K531 site. Lys, lysine; Arg, arginine; Glu, glutamate. (E and F) K531R, K531E site mutations, or wild-type Flag-ATP5F1A-overexpressed HAVSMCs were constructed respectively through overexpression plasmid. Flag-ATP5F1A-overexpressed HAVSMCs proteins were enriched by Flag antibody and detected with anti-lactyllysine antibody (*n* = 3). (G) Cell migrations of HAVSMCs following the ATP5F1A K531 site mutation with Nala treatment were detected using crystal violet dye solution. Scale bar, 500 μm. (H) Detection of apoptosis in HAVSMCs after the ATP5F1A K531 site mutation with Nala treatment using TUNEL staining. Scale bar, 500 μm. (I to L) Western Blots was conducted to measure the protein expression level of phenotypic switch markers (I) and MMP-2/9 (K) after ATP5F1A K531 site mutation with Nala treatment (*n* = 3), OPN, osteopontin. Data are means ± SEM from 3 independent experiments and were analyzed by Kruskal–Wallis test with Dunn post hoc test (B, C, F, J, and L). **P < 0.01, ***P < 0.001, and ****P < 0.0001.

To further clarify the modification of ATP5F1A, we used co-immunoprecipitation (Co-IP) to verify its lactylation level. The lactylation of ATP5F1A was found to increase upon exogenous lactate treatment in HAVSMCs (Fig. 5A and B). In contrast, the expression of ATP5F1A in whole-cell lysates decreased after Nala treatment (Fig. 5C). To verify the effect of ATP5F1A lactylation on mitochondrial and cellular functions of HAVSMCs, we subjected ATP5F1A to site-specific mutations. Recombinant plasmids (with green fluorescent protein) were used to transfect HAVSMCs with mutated ATP5F1A, in which K531 was replaced by arginine or glutamate, thereby generating de-Kla (K531R) or Kla-mimicking (K531E) mutant, respectively (Fig. 5D and Fig. S4). IP was performed to verify Kla levels in ATP5AF1A (Fig. 5E and F). Overexpression of wild-type or site-mutated ATP5F1A incorporated into mitochondria had no notable effect on the viability of HAVSMCs. In the presence of Nala, the survival rate of HAVSMCs overexpressing the ATP5F1A K531R mutant was similar to that of the control group. In the presence of Nala, ATP5F1A K531R inhibited cellular migration, whereas ATP5F1A K531E promoted it (Fig. 5G). Surprisingly, this was not observed without lactate. Transwell assays showed that ATP5F1A K531R mutations promoted the migration of HAVSMCs, whereas ATP5F1A K531E mutations suppressed it (Fig. S5A). We speculated that this discrepancy could be attributed to nonlactylation pathways involved in lactate production that may also influence HAVSMC migration. Under physiological lactate, K531R enhances ATP production, powering cytoskeletal dynamics for migration; K531E impairs energy supply, inhibiting motility. Under exogenous lactate treatment (25 mM), although the K531 site of ATP5F1A retains its regulatory role in cell migration, lactate can promote VSMC migration through nonlactylation pathways [27–29]. Consequently, the promigratory effect of lactate may partially override K531-lactylation-dependent regulation.

Fluorescent labeling of the deoxyuridine triphosphate (dUTP) notch end of terminal deoxynucleotide transferase showed that ATP5F1A K531E mutant have no effect on apoptosis of HAVSMCs (Fig. 5H and Fig. S5B).

Furthermore, we observed that this finding differed from the results obtained with drug treatments, suggesting that the alteration in HAVSMC apoptosis is not due to a general change in lactylation but rather specifically the lactylation of ATP5F1A. The use of drugs alone did not notably alter the lactylation level of ATP5F1A. In addition, Western blotting showed that K531E mutation reduced the expression of contractile phenotype marker proteins and matrix metalloenzymes in HAVSMCs (Fig. 5I to L and Fig. S5C and D), indicating that the lactylation modification of ATP5F1A plays a crucial role in modulating the function of HAVSMCs.

### Lactylation of ATP5F1A may affect mitochondria by regulating ATP5F1A expression

**Fig. 6.**
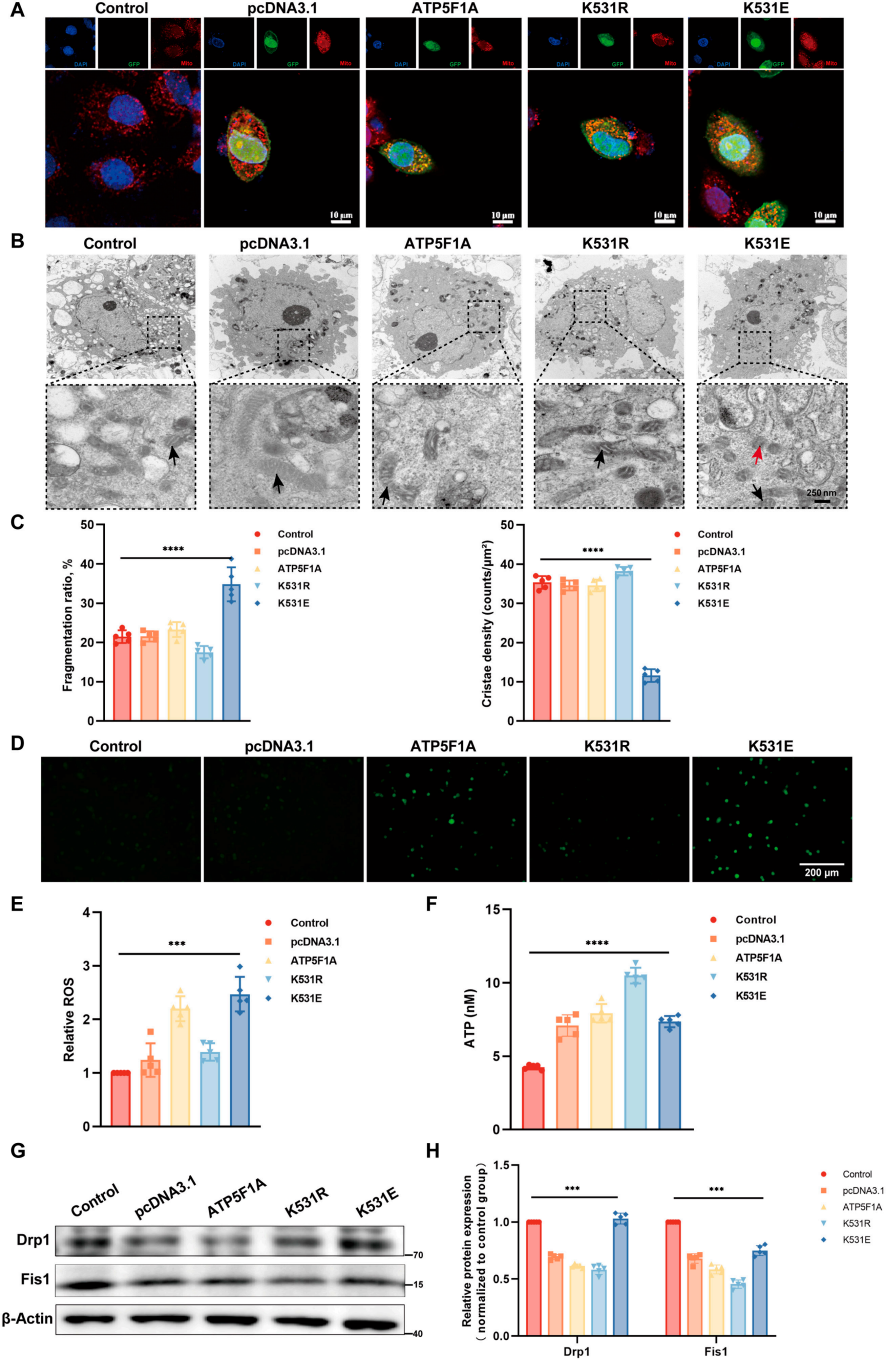
Lactylation of ATP5F1A at K531 affects mitochondrial function. Wild-type, K531R, or K531E site mutation ATP5F1A-overexpressed HAVSMCs were constructed through the pcDNA3.1 vector. (A) Mitochondrial tracing using mitochondria-specific fluorescent probes. Scale bars, 10 μm. GFP, green fluorescent protein. (B) Mitochondrial structure observed by mitochondrial scanning electron microscopy. Scale bar, 250 nm. Black arrows indicate mitochondria, and red arrows indicate broken mitochondria. (C) Left: Mitochondrial fragmentation ratio (proportion of mitochondria with long axis < 2 μm). Right: Number of mitochondrial cristae per unit area. (D and E) Detection of cellular ROS production by dihydrofluorescein diacetate (DCFH-DA) fluorescent staining (*n* = 3). Scale bar, 200 μm. (F) ATP production assay (*n* = 5). (G and H) Western Blots was conducted to measure the protein expression level of mitochondrial division (*n* = 3) Fis1, mitochondrial fission 1 protein. Data are means ± SEM from 3 independent experiments and were analyzed by Kruskal–Wallis test with Dunn post hoc test (D, E, and G). ***P < 0.001 and ****P < 0.0001.

ATP5F1A is mainly expressed in the inner mitochondrial membrane and is essential for maintaining mitochondrial function [30]. We found that K531E mutation in ATP5F1A reduced mature mitochondrial number (Fig. 6A). In addition, mitochondrial structure was found to be abnormal when K531E-mutated ATP5F1A was overexpressed in HAVSMCs (Fig. 6B), including an increased proportion of mitochondrial fragments and decreased cristae density of mitochondria (Fig. 6C). Therefore, we determined whether lactylation modification at K531 of ATP5F1A affects mitochondrial morphology, number, and production of reactive oxygen species (ROS) in HAVSMCs (Fig. 6D). The results showed that ATP5F1A K531E mutation increased mitochondrial fission (Fig. 6B and C), increased the production of ROS (Fig. 6D and E), and reduced ATP production (Fig. 6F). Consistent with this, the expression level of mitochondrial fission marker proteins increased in K531E (Fig. 6G and H). Subsequently, K531E mutation of ATP5F1A increased ROS production, and this increase was more notable in the presence of Nala (Fig. S5E). After HAVSMCs were treated with Nala at different concentrations, ATP5F1A expression gradually decreased (Fig. S6A), while the mRNA level remained unchanged (Fig. S6C). To investigate whether the decrease in ATP5F1A expression was due to protein lactylation, FLAG-ATP5F1A was overexpressed in HAVSMCs treated with Nala. The results showed that expression of exogenous ATP5F1A decreased after lactate treatment (Fig. S6B). However, we transfected HAVSMCs with plasmids expressing wild-type and mutant ATP5F1A. No significant difference was noted in mRNA expression of ATP5F1A between the wild-type and mutant groups (Fig. S6D). In addition, we transfected 293T cells with plasmids expressing wild-type or K531R ATP5F1A, followed by cycloheximide treatment to inhibit protein synthesis. The results revealed that K531R delayed ATP5F1A degradation (Fig. S6E). These findings suggest that lactate can reduce ATP5F1A expression, and this reduction is mediated through the lactylation of ATP5F1A, which, in turn, affects its protein degradation.

### Sirt3 serves as the lactylation eraser for ATP5F1A

**Fig. 7.**
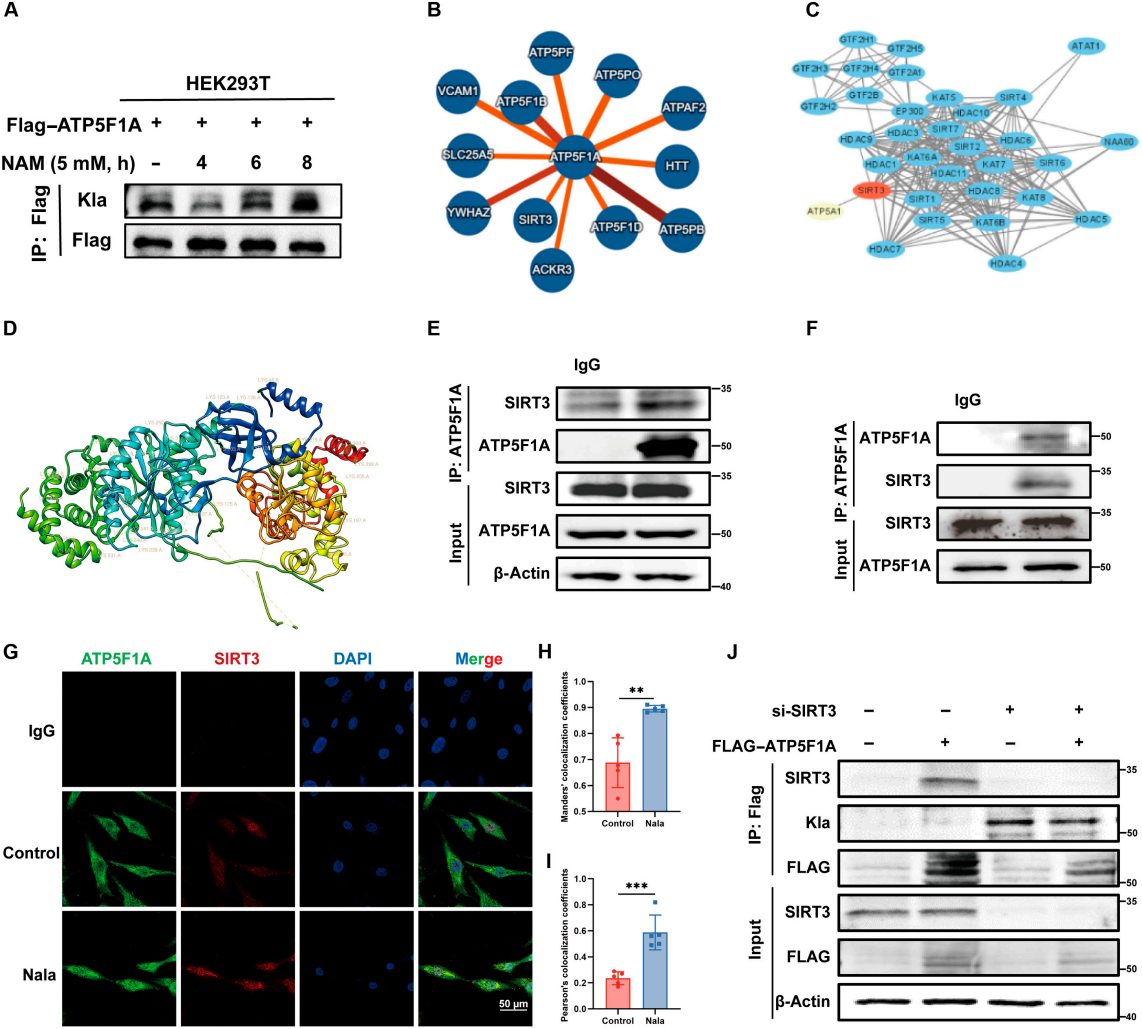
Sirt3 regulates the lactylation of ATP5F1A. (A) Flag-ATP5F1A transfection of 293T cells was performed to achieve ATP5F1A overexpression, followed by treatment with NAM (5 mM). Western blots were used to detect Flag expression level. (B) Predicted force directed top 12 proteins that bind to ATP5F1A. (C) Prediction of the binding of acetylated enzymes to ATP5F1A. (D) Predicted 3D structure of the Sirt3–ATP5F1A complex. (E) Co-IP shows that Sirt3 can specifically bind to ATP5F1A. (F) Co-IP shows that ATP5F1A can specifically bind to Sirt3. (G) Immunofluorescence colocalization shows overlapping distribution of Sirt3 and ATP5F1A on mitochondria. Scale bar, 50 μm. (H and I) Characterization of colocalization using Manders’ colocalization coefficient and Pearson’s correlation coefficient (*n* = 5). (J) 293T cells were cotransfected with si-Sirt3 and FLAG-tagged overexpression vector for ATP5F1A, and the level of ATP5F1A lactylation was assessed. Data are means ± SEM from 3 independent experiments and were analyzed by Student’s *t* test (I and J). **P < 0.01 and ***P < 0.001.

Niacinamide, a sirtuin family inhibitor, was used to treat HAVSMCs, and an increase in the lactylation level of ATP5F1A was observed (Fig. 7A). To identify the enzymes that regulate lactate modification of ATP5F1A, we used the online software IntAct for predicting the binding of ATP5F1A (https://www.ebi.ac.uk/intact/search?query=ENSG00000152234&expanded=true&layout=avsdf). The results showed that 317 human proteins bound to ATP5F1A (Fig. S7A), among which the top 12 proteins were force-directed (Fig. 7B), of which Sirt3 aroused our interest. The sirtuin family contains 7 members, silencing information regulatory factor 2 related enzymes (SIRT1 to SIRT7), which have different intracellular locations. Sirt3 is the only deacetylase located in the mitochondria [31], and it has a good correlation with ATP5F1A in the organization (Fig. S7B). Still, we found that Sirt3 does not affect the protein expression level of ATP5F1A under physiological conditions (Fig. S7C). The role of Sirt3 as a delactating enzyme in tumors has been reported [32]; however, its role in AD has not been studied. In addition, we will also analyze the reported acetylation regulatory enzymes binding to ATP5F1A on STRING website (Fig. 7C), and only Sirt3 shows the possibility of binding. We analyzed the correlation between Sirt3 and ATP5F1A expression in human tissues using the Genotype Tissue Expression database. Therefore, we conducted a molecular docking study to evaluate the binding mode of ATP5F1A and Sirt3 using ZDOCK and Chimera software (Fig. 7D). Co-IP experiments were used to verify the binding of Sirt3 and ATP5F1A, and mutual binding was observed between these 2 proteins (Fig. 7E and F). Similar results were obtained via immunofluorescence colocalization, and Sirt3 and ATP5F1A had overlapping distributions in the mitochondria (Fig. 7G), with quantification of Manders’ colocalization coefficients and Pearson’s colocalization coefficients (Fig. 7H and I). To further clarify the interaction between Sirt3 and ATP5F1A and role of Sirt3 on the regulation of ATP5F1A lactylation, we synthesized a small interfering RNA (siRNA) of Sirt3 and transfected it into human embryonic kidney (HEK) 293T cells that also overexpressed Flag-ATP5F1A. Co-IP studies showed that the binding of ATP5F1A and Sirt3 and the degree of Kla of ATP5F1A increased when Sirt3 was inhibited (Fig. 7J). Therefore, we conclude that Sirt3 is a lactylation eraser of ATP5F1A.

### ATP5F1A K531 lactylation promotes the development of AD in vivo

**Fig. 8.**
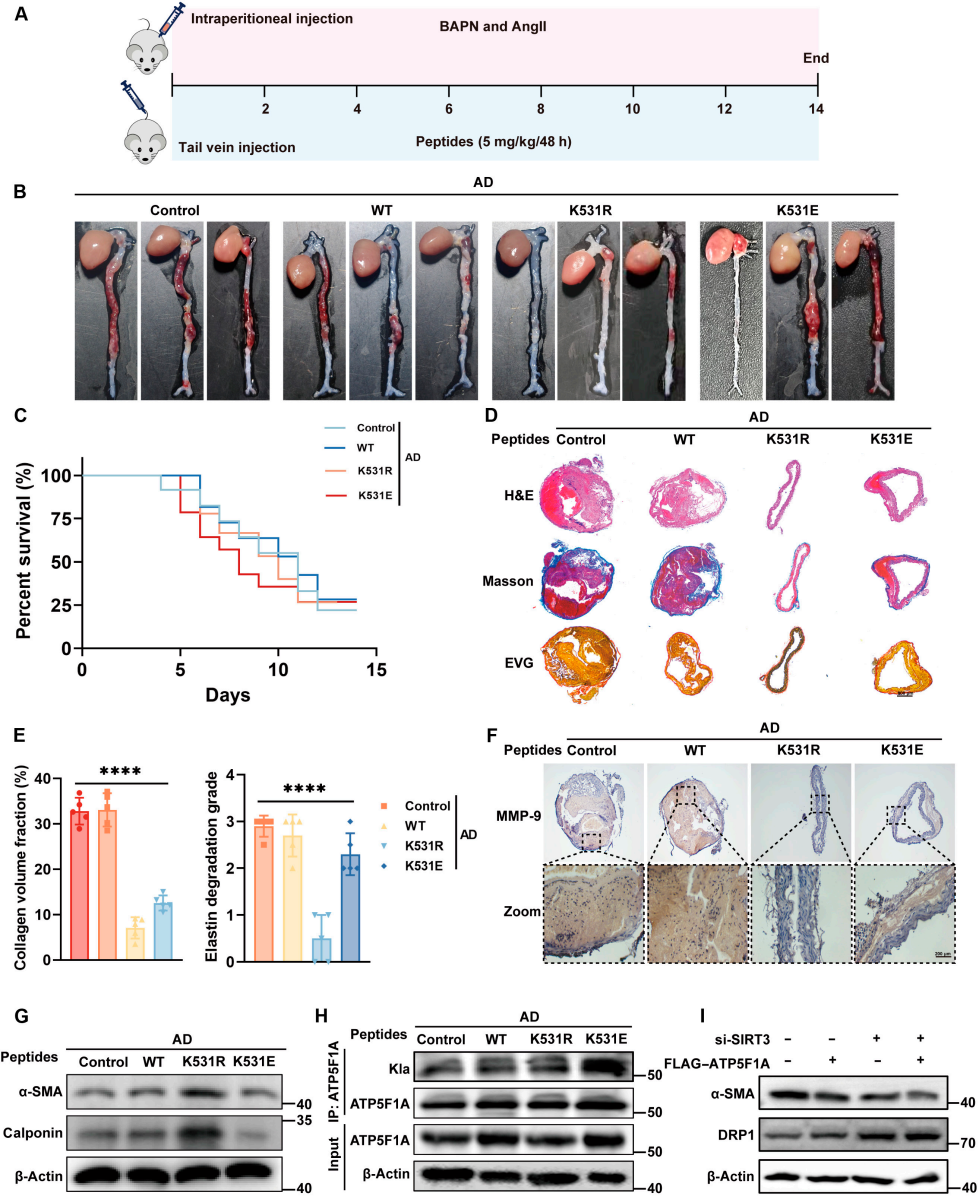
Effect of ATP5F1A K531 lactylation on aortic dissection in vivo. (A) AD mice were constructed by injecting BAPN and AngII. K531R peptides and K531E peptides were injected into the tail vein for 14 days (*n* = 11). (B) Typical images showed macroscopic features of isolated mice aorta from different groups (*n* = 3). (C) Survival rate curve of each group of mice. WT, wild-type. (D and E) Representative images of H&E, EVG, and Masson staining in thoracic arteries obtained from mice in different groups (*n* = 5). Scale bar, 800 μm. (F) Immunohistochemistry of MMP-9 in different groups. Detection of LDHA expression levels in different groups. (G) Western blot was used to detect the marker of contractile phenotype mice in different groups. (H) Co-IP was used to detect the ATP5F1A lactylation level of the aorta of mice in different groups. (I) Western blot was used to detect the marker of contractile phenotype and mitochondrial division. Data are means ± SEM from 3 or 5 independent experiments. ****P < 0.0001.

To investigate the effect of the ATP5F1A K531 mutation on AD progression in mice, we successfully generated ATP5F1A K531 site mutant mice using intraperitoneal injection of modified peptides (Fig. 8A). Co-IP analysis revealed a significant increase in ATP5F1A lactylation levels in K531E mutant mice, while a marked decrease in lactylation was observed in K531R mutant mice (Fig. 8H). Our study found that the K531R mutation significantly reduced the incidence of AD and prolonged the survival of mice, whereas the K531E mutation increased AD incidence and shortened survival (Fig. 8B and C). Furthermore, K531E-treated mice exhibited more severe elastic fiber degradation and interstitial fibrosis deposition, while K531R treatment alleviated these pathological changes (Fig. 8D and E). K531E treatment inhibited the expression of contractile phenotype proteins and promoted elevated levels of MMP-9, whereas K531R treatment had the opposite effect (Fig. 8F and G). In addition, we knocked down Sirt3 and overexpressed ATP5F1A in HAVSMC to detect cell function. Consistent with previous findings using mutants, knocking down Sirt3 led to an increase in the lactate level of ATP5F1A, which resulted in a decrease in the expression of contractile phenotype markers and an increase in the expression level of mitochondrial division inhibitor 1 (DRP1) (Fig. 8I). Therefore, ATP5F1A K531 lactylation plays a facilitating role in the pathological progression of AD.

## Discussion

**Fig. 9.**
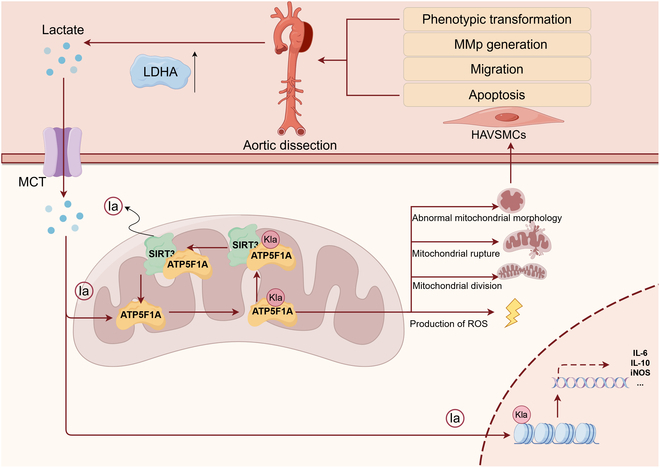
Sirt3 regulates the lactylation of ATP5F1A in mitochondria and affects the progression of aortic dissection. The increased expression of LDHA in aortic dissection leads to an increase in lactate content in circulation and tissues. Lactate enters mitochondria and nuclei in the form of lactoyl groups, causing protein to undergo lactylation modification. The lactylation modification of ATP5F1A regulated by Sirt3 in the mitochondria of HAVSMCs affects the function of ATPase, resulting in mitochondrial damage, rupture, functional abnormalities, reduced ATP production, increased ROS production, and, ultimately, an increase in the number of synthetic HAVSMCs and the secretion of MMPs, thereby aggravating aortic dissection. MCT, monocarboxylate transporter; IL-6, interleukin-6; iNOS, inducible nitric oxide synthase.

The first 2 weeks of AD are considered to be the acute phase, during which patients are highly susceptible to life-threatening complications and mortality; therefore, rapid diagnosis and appropriate treatment strategies are critical for managing the affected patients. Here, we noticed that ATP5F1A lactylation regulates vascular remodeling and promotes the progression of AD. Specifically, we pinpointed lactylation sites and observed an increase in lactylation at K531 of ATP5F1A during AD. This is the first study to detect aortic lactylation during both physiological and pathological processes. In our study, we found that lactylation was increased during aortic dissection, and the function of lactylation at the lysine-531 site of ATP5F1A in HAVSMCs was verified in detail. This indicates that lactylation plays an important role in the development of AD. It is worth noting that the lactylation of ATP5F1A may affects the protein expression level of ATP5F1A, resulting in the function of ATP synthase in the mitochondria being affected, ATP synthesis being reduced, and mitochondrial morphology being altered, thus promoting the synthetic phenotype of HAVSMCs (Fig. 9).

LDHA is closely associated with the occurrence of cardiovascular diseases [33,34]. LDHA has been studied as a therapeutic target for diseases such as heart failure, atherosclerosis, and hypertension [34–36]. In addition, LDHA participates in the proliferation and migration of HAVSMCs [37] and regulates MMP expression in tumors [38]. Previous studies have shown that LDHA expression is up-regulated in AD, both at the RNA and protein levels [34]. Lactate is the end product of glycolysis that involves LDHA. In this study, we treated HAVSMCs with endogenous lactate production regulatory drugs and directly treated them with exogenous lactate. The results showed that when lactate production increased in HAVSMCs, the migration of HAVSMCS changed, the synthetic phenotype increased, the production of MMP-2 and MMP-9 elevated, and apoptosis was also slightly affected. While we used an LDHA inhibitor to study the effect of lactylation on aortic dissection in vivo, it is important to note that LDHA inhibition reduces lactate production, potentially lowering overall aortic lactylation levels. However, LDHA also mediates aerobic glycolysis, and inhibiting LDHA can suppress glycolysis, which may affect VSMC phenotypic transformation and aortic dissection through other mechanisms [39]. We acknowledge that FX-11, as an LDHA inhibitor, may indirectly affect lactylation modification by reducing lactate production through glycolysis inhibition. However, this study supports the direct role of lactylation through validation in the K531 mutation model, where reduced lactylation levels and improved AD phenotypes were closely aligned in both the FX-11-treated group and the ATP5F1A K531R mutant model. This indicates that phenotypic improvements primarily arise from lactylation inhibition rather than global modulation of the glycolytic pathway. Therefore, the protective effect of the LDHA inhibitor on aortic dissection cannot be solely attributed to reduced lactylation levels. However, there is no doubt that the changes in lactylation modification mediated by LDHA inhibition play a critical role in VSMC phenotypic transformation and the pathogenesis of AD.

ATP5F1A is a crucial subunit of ATP synthase in mitochondria, primarily responsible for ATP synthesis [30]. Mutation or deletion in ATP5F1A may lead to serious mitochondrial diseases and pose a considerable threat to the motor and nervous systems. In addition, phosphorylation of ATP5F1A at Y243/246 can maintain high mitochondrial output, and this mechanism is activated in prostate cancer [40], suggesting that posttranslational modification of ATP5F1A has a potentially powerful regulatory role in diseases. Our study identified a novel posttranslational modification of ATP5F1A and revealed the role of lactylation of ATP5F1A in the progression of AD, while exploring the enzymes that regulate this process.

As a member of the sirtuin family, Sirt3 is involved in various cellular processes through deacetylation, including cell growth, survival, apoptosis, aging, metabolism, and maintenance of mitochondrial integrity [41–43]. Although Sirt3 has been shown to improve mitochondrial function by deacetylating mitochondrial proteins, enhancing autophagy, and inhibiting myocardial hypertrophy [44,45], the specific molecular mechanisms are still not fully understood. Under physiological conditions, Sirt3 knockdown does not alter ATP5F1A protein expression (Fig. S7C). This is safeguarded by 2 protective mechanisms: First, basal ATP5F1A expression is primarily regulated by nuclear respiratory factor 1 (NRF1) and peroxisome-proliferator-activated receptor γ [46,47], indicating that its promoter activity should be independent of SIRT3. Second, in the physiologically low-lactate environment, ATP5F1A lactylation levels are minimal, and its protein stability does not require maintenance by Sirt’s delactylase activity. Conversely, in pathological hyperlactate environments, K531 lactylation modification of ATP5F1A accumulates significantly, potentially accelerating its degradation via the proteasomal pathway (Fig. S6E). Here, SIRT3’s critical role emerges—acting as an essential “lactylation buffer”, its delactylase activity blocks K531 lactylation, thereby maintaining ATP5F1A stability. This mechanism explains why SIRT3 becomes indispensable for ATP5F1A stability only during lactate overload and provides a precise theoretical foundation for targeting the SIRT3–lactylation axis to treat diseases (e.g., aortic dissection). Although Sirt3 deacetylation activity has been demonstrated and thousands of substrates have been examined by multitissue quantitative proteomics, bioinformatics analysis, and studies on biochemical effectiveness, very few functions of Sirt3 are associated with cardiovascular disease. Our study discovered a novel role for Sirt3 in the delactylation of ATP5F1A, a finding that has not been previously reported. This suggests that Sirt3 may be involved in aortic dissection (AD), warranting further investigation. To explore this possibility, we examined whether Sirt3 contributes to the lactylation of ATP5F1A in AD. Previous reports have indicated that Sirt1 and histone deacetylase 1 (HDAC1) to HDAC3 function as lactylation erasers [48,49]. Among them, HDAC1 and Sirt1 are associated with AD [50–52]; therefore, they may modulate lactylation in AD. Nevertheless, the relationship between acetylation and lactylation is currently unclear, given the regulatory effects of HDAC1 and Sirt1 on acetylation. At present, there is still insufficient research on lactylation modification and its key catalytic enzymes. Therefore, we are currently unable to reach a definitive conclusion, and further studies are required.

As key posttranslational modifications, lactylation and acetylation have been reported to form a dynamic interaction network through shared target proteins [53–55], competitive modification [56], and shared regulatory enzymes (e.g., p300 and Sirt1/Sirt3) [49,57]. This network synergistically regulates metabolic reprogramming, epigenetic remodeling, and cellular functional homeostasis, providing new integrated perspectives for understanding disease mechanisms. Notably, studies indicate that Sirt3 possesses both deacetylase and delactylase activities, suggesting its potential dual regulatory role within the metabolic modification network [57,58]. Although this study did not directly assess ATP5F1A acetylation levels, our proteomic analysis revealed significantly elevated lactylation levels of the mitochondrial metabolic enzyme phosphoglycerate kinase 1 (PGK1) in Alzheimer’s disease tissues. Given that PGK1 is also regulated by acetylation, we speculate that lactylation may interfere with mitochondrial metabolic enzyme activity via cross-talk with acetylation, thereby potentially influencing AD progression. Systematic elucidation of the lactylation–acetylation interaction network in AD, however, requires further investigation using dual-omics (lactylome and acetylome) coanalysis.

In summary, we provided an overview of lactylation in AD, identified the adverse effects of lactate and lactylation on AD, and screened and validated the regulatory mechanism of ATP5F1A K531 lactylation on function of HAVSMCs. We broadened the clinical control and treatment options of AD and identified new potential cell therapy targets from the perspective of posttranslational modification. In addition, the regulatory enzymes of protein modification and their key roles in diseases are also worth further explore.

## Materials and Methods

### Collection of AD tissues

AD tissue specimens were obtained from patients undergoing aortic resection at the Affiliated Hospital of Qingdao University (Qingdao, Shandong, China), and the normal aortic samples were collected from adjacent tissue areas of patients with gastrointestinal tumors, specifically from the mesenteric arteries. This study has been recognized by the Medical Ethics Committee of Qingdao University Affiliated Hospital (no. QYFY WZLL 28347). The investigation conformed to the principles outlined in the Declaration of Helsinki. The clinical inclusion criteria for this study were as follows: patients aged ≥18 years, individuals with clinically suspected symptoms indicative of AD, those with imaging evidence confirming AD, and those who were willing and capable of providing written informed consent. The exclusion criteria comprised unwillingness or inability to provide informed consent, individuals below 18 years of age, patients diagnosed with cardiovascular conditions mimicking AD, those with known hereditary disorders such as Ehlers–Danlos syndrome, Loeys–Dietz syndrome, or Marfan syndrome, and individuals with a history of surgical repair for AD. The tissues were cryopreserved in liquid nitrogen for further use. Ethical approval for the study was obtained from the Affiliated Hospital of Qingdao University, and signed informed consent was obtained from all participating patients. Detailed information about the clinical samples is provided in Table S1.

### Animal models

All animal studies were approved by the Experimental Animal Ethics Committee of the Medical Department of Qingdao University (Shandong, China) and were in accordance with the National Institutes of Health Guide for the Care and Use of Laboratory Animals. The animal models were generated as previously described, with slight modifications [20,59,60]. All animal experiments were approved by the Animal Care and Use Committee at the Experimental Animal Center of Qingdao University, China. Male C57BL/6 mice (3-week-old) were purchased from Vital River Laboratory Animal Technology Co. Ltd. (Beijing, China). Mice were divided into control (*n* = 11), AD (*n* = 11), AD + phosphate-buffered saline (PBS) (*n* = 11), AD + Nala (*n* = 11), and AD + FX-11 (*n*=11) groups. PBS, Nala (1 g/kg), and FX-11 (4 mg/kg) were intraperitoneally injected once a day for 7 days. After 3 days, saline (200 μl) was intraperitoneally injected every 8 h in the control group, whereas the other groups of mice were intraperitoneally injected with AngII (4 mg/kg) (GL Biochem, Shanghai, China) every 8 h and BAPN (0.33 g/kg) (Aladdin, Shanghai, China) every 24 h. Mice were euthanized, and aortic tissues were collected. Euthanasia was performed with ketamine (100 mg/kg body weight) and thiazine (20 mg/kg body weight) under anesthesia, followed by exsanguination.

### Cell culture

HAVSMCs were purchased from the American Type Culture Collection cell library and cultured in Dulbecco's modified Eagle's medium (DMEM) supplemented with 10% fetal bovine serum (FBS). The cells were maintained at 37 °C in a 5% CO_2_ environment and regularly subcultured until the logarithmic growth phase.

### Protein extraction and trypsin digestion

The samples underwent cryogenic grinding to form a fine powder using liquid nitrogen. Each sample group was then individually combined with lysis buffer (comprising 1% SDS, 1% protease inhibitor, 3 μM trichostatin A, and 50 mM nicotinamide) at a 1:4 ratio, followed by sonication to extract the supernatant. Equal amounts of protein were retrieved from each sample, and the volume was standardized using the lysis buffer. For protein precipitation, an equivalent volume of prechilled acetone was added, followed by thorough vortex mixing. An additional 4-fold volume of prechilled acetone was introduced. The resulting mixture was allowed to precipitate at −20 °C for 2 h with centrifugation at 4,500*g*. Afterward, the supernatant was discarded, and the precipitate underwent 2 rounds of washing with prechilled acetone before being air-dried. To dissolve the dried precipitate, 200 mM tetraethylammonium bromide was added, followed by sonication. Trypsin was then added to the mixture at a 1:50 ratio, and an overnight digestion process was carried out. To achieve a final concentration of 5 mM, dithiothreitol was introduced, and the mixture was reduced at 56 °C for 30 min. Subsequently, iodoacetamide was added to reach a final concentration of 11 mM, and the mixture was incubated at room temperature in a light-protected environment for 15 min.

### Modification enrichment

The peptide fragments were solubilized in an IP buffer solution composed of 100 mM NaCl, 1 mM EDTA, 50 mM tris-HCl, and 0.5% NP-40 and adjusted to pH 8.0. The resulting supernatant was then applied onto prewashed lactylated resin (PTM-1404, Jingjie, Hangzhou). The resin was gently agitated on a rotating shaker at 4 °C for an overnight incubation. Following the incubation, the resin underwent 4 washes with the IP buffer solution, followed by 2 rinses with deionized water. Subsequently, elution of the bound peptide segments was carried out using a wash solution containing 0.1% trifluoroacetic acid. This elution process was repeated 3 times, and the resulting eluate was collected and subjected to vacuum freeze-drying. After the drying step, desalting was performed following the instructions outlined in the C18 ZipTips manual. Once desalted, the samples were prepared for subsequent analysis using liquid chromatography (LC)-MS.

### LC-MS analysis

Peptide fragments were solubilized in mobile phase A, which consisted of an aqueous solution of 0.1% formic acid and 2% acetonitrile. Subsequently, the fragments were separated using a highly efficient NanoElute ultrahigh-performance LC (UHPLC) system. Peptides were eluted using mobile phase B, which consisted of a solution containing 0.1% formic acid and 100% acetonitrile. The chromatographic gradient followed the specific program mentioned here: 0 to 42 min, a gradual and linear increase in the concentration of mobile phase B from 7% to 24%; between 42 and 54 min, a continuous linear increase from 24% to 32% of B; between 54 and 57 min, linear increase in concentration from 32% to 80% of B; and between 57 and 60 min, isocratic elution maintained with 80% B. The flow rate was maintained at 450 nl/min throughout the analysis. After UHPLC separation, the peptide fragments were introduced into the capillary ion source for ionization, followed by analysis using a PRO mass spectrometer. The ionization voltage applied at the ion source was set to 1.75 kV. Both precursor ions and their corresponding fragmentation products were detected and analyzed using a high-resolution time-of-flight detection system. The mass spectrometer was operated within the mass/charge ratio range of 400 to 1,500 to acquire MS/MS scans. Data were acquired using the parallel accumulation-serial fragmentation (PASEF) mode. After obtaining a full MS spectrum, a total of 10 successive rounds of PASEF MS/MS scans was meticulously executed, specifically targeting precursor ions with charge states ranging from 0 to 5. This approach effectively minimized redundant scanning of identical precursor ions by implementing a dynamic exclusion time of 30 s during MS/MS scans.

### Western blot

The sample was lysed in an ice bath for at least 15 min using protein lysis buffer (Solarbio, Beijing, China). The resulting lysate was collected as the supernatant, and protein quantification was carried out following the manufacturer’s guidelines (Solarbio, Beijing, China). Following this, the protein sample was mixed with loading buffer and heated to 95 °C for 10 min. The protein was then separated by electrophoresis using a 10% SDS-polyacrylamide gel and subsequently transferred onto a 0.45-μm polyvinylidene fluoride membrane. The membrane was subsequently blocked with a 5% milk solution in tris-buffered saline with Tween 20 (TBST) at room temperature for 1 h. The primary antibody was diluted in 5% bovine serum albumin (BSA) in TBST and incubated with the membrane overnight at 4 °C. Following this, the corresponding secondary antibody, also diluted in 5% BSA in TBST, was incubated at room temperature for 1 h. Chemiluminescence was used for image visualization (ProteinSimple, CA, USA), and quantitative analysis was conducted using ImageJ 1.8.0 software. The primary and secondary antibodies were diluted as follows: rabbit anti-α-SMA (Abcam; 1:1,000), rabbit anti-calponin 1 (Abcam; 1:1,000); rabbit antimyosin heavy polypeptide 11 (Abcam; 1:1,000); mouse anti-ATP synthase, H^+^-transporting, mitochondrial F1 complex, α subunit 1, cardiac muscle (ATP5A1; Proteintech; 1:5,000); rabbit anti-lactyl-histone H3 monoclonal antibody (Kla; PTM BIO; 1:2,000); rabbit anti-β-actin monoclonal antibody (ABclonal; 1:100,000); goat anti-mouse immunoglobulin G (IgG)–horseradish peroxidase (HRP) (absin; 1:5,000); goat anti-rabbit IgG–HRP (absin; 1:5,000); peroxidase-conjugated IgG fraction monoclonal mouse anti-rabbit IgG, light chain specific (the Jackson Laboratory; 1:10,000).

### Immunohistochemistry

After dewaxing with xylene, slices were boiled for antigen retrieval. The slices were then incubated with hydrogen peroxide for 10 to 15 min to reduce nonspecific background staining caused by endogenous peroxidases. The tissues were covered with a working solution of primary antibody against Kla (dilution, 1:2,000; PTM BIO, Hangzhou, Zhejiang, China) and incubated overnight at 4 °C. The slices were then incubated with an anti-rabbit antibody for immunohistochemistry (ZSGB-BIO, Beijing, China) and incubated for 30 min at 25 °C. Subsequently, the tissues were stained with hematoxylin and 3,3′-diaminobenzidine. Finally, the slices were mounted using a cover glass, observed, and photographed using a microscope (Nikon, Japan).

### RNA extraction and quantitative real-time polymerase chain reaction

Following the manufacturer’s instructions, total RNA from cell and tissue samples was purified using Trizol (Sigma, Louis, MO, USA). Subsequently, cDNA synthesis was carried out using a reverse transcriptase kit (Takara, Kyoto, Japan). To quantify RNA expression, real-time quantitative polymerase chain reaction (PCR) was conducted using SYBR Green PCR Master Mix (Yeasen, Shanghai, China). The expression level of target genes was standardized using the housekeeping gene glyceraldehyde-3-phosphate dehydrogenase. Primer refers to Table S2 for the primer sequences.

### Transwell assay

Initially, cells were seeded in 12-well plates and allowed to proliferate until reaching 70% to 80% confluence before undergoing treatment (drug or plasmid transfection) for 24 h. Subsequently, cells were detached using trypsin, and approximately 3 × 10^4^ cells were then individually seeded into Transwell upper chambers with 0.8-μm pores, positioned within a 24-well plate (Corning, USA). The upper chambers were filled with 200 μl of DMEM without FBS, while the lower chambers were loaded with 500 μl of DMEM containing 10% FBS. Following 24 h of incubation for migration, cells were fixed with 4% paraformaldehyde for 30 min, followed by staining with 0.1% crystal violet for 30 min. Finally, 5 fields within each membrane were observed and quantified under a microscope (Nikon, Japan).

### Terminal deoxynucleotidyl transferase dUTP nick end labeling assay

Cells were cultured in 48-well plates and subjected to a 24-h transfection period. Then, cells were fixed for 30 min using 4% paraformaldehyde and permeabilized using 0.5% Triton X-100. A terminal deoxynucleotidyl transferase dUTP nick end labeling (TUNEL) Apoptosis Assay Kit (Yeasen Biotechnology Co. Ltd., Shanghai, China) was used according to the manufacturer’s instructions. TUNEL solution was applied to cells and incubated at 37 °C for 1 h. Finally, slides were mounted using an antifade mounting medium suitable for fluorescence microscopy. Cells were observed using a fluorescence microscope (Nikon), and images of 5 different fields of view were captured.

### Cell proliferation assay

Cells were inoculated into 96-well plates, and after 24 h of transfection, 10 μl of Cell Counting Kit-8 (CCK-8) reagent (Yeasen, Shanghai, China) were added to each well and incubated at 37 °C for 1 h. Subsequently, an enzyme-linked immunosorbent assay reader was used to detect absorbance at 450 nm (Thermo Fisher Scientific, Milan, Italy).

### Immunofluorescence

For immunofluorescence, the tissues were dehydrated and embedded in paraffin and cut into 6-μl slices. Slices were blocked with 5% goat serum for 30 min and incubated overnight at 4 °C using the first antibody: anti-Kla (PTM BIO, China), anti-ATP5A1 (Proteintech, China), and anti-α-SMA (Abcam, China). Then, they were incubated with the secondary antibodies for 1 h. Cell nuclei were counterstained with 4′,6-diamidino-2-phenylindole (DAPI; Beyotime, China). Isotype controls were performed using rabbit IgG (ab172730, Abcam, USA) and mouse IgG (ab18413, Abcam, USA) at the same concentrations as the primary antibodies. Secondary antibody controls were conducted to distinguish genuine target staining from the background. For the results of colocalization, use ImageJ for Manders’ colocalization coefficient and Pearson’s colocalization coefficient analyses.

### Co-IP assay

Whole-cell extracts were lysed in radioimmunoprecipitation assay lysis buffer (Solarbio, Beijing, China) containing a mixture of protease inhibitors (Solarbio, Beijing, China), deacetylase inhibitors (Abmole Bioscience, Shanghai, China), and phenylmethylsulphonyl fluoride (Solarbio, Beijing, China). Cell lysates were centrifuged at 12,000*g* for 15 min, and the supernatant was collected and incubated with protein A/G magnetic beads (MedChemExpress, Shanghai, China) bound to specific antibodies. After incubation overnight at 4 °C on a rotary mixer, the magnetic beads were washed 5 times with IP washing buffer, and proteins were eluted using Laemmli buffer for further detection.

### Quantification of l-lactate

Following the manufacturer’s instructions, the lactate colorimetric assay kit (E-BC-K044-M, Elabscience) was used to measure the intracellular lactate content. Briefly, 5 × 10^5^ VSMCs treated with different drugs were collected. After removed the culture medium, the cells was washed twice with precooled PBS. Subsequently, 200 μl of PBS was added to scrape the cells and homogenized on ice using an ultrasonic crusher. Following homogenization, the samples were centrifuged at 12,000*g* for 10 min at 4 °C. The resulting supernatant was utilized for the lactate measurement. Finally, the lactate content was normalized to its corresponding protein content.

### ATP generation assay

A Cell Titre-Glo Luminescent Cell Viability Assay Kit (G7570, Promega, Beijing, China) was used to quantify the cellular and mitochondrial ATP levels. Cells were grown in 96-well plates for 24 h, and 100 μl of ATP reagent was added to each well and mixed thoroughly. The plates were then incubated at room temperature in the dark for 10 min, and luminescence was measured using a photometer. For each experiment, the values were normalized to those of the control group.

### ROS determination

Cells were grown in 24-well plates, treated, and incubated with 2,7-dichlorodihydrofluorescein diacetate. Cells were then washed thrice with serum-free medium and observed using a fluorescence microscope (Nikon), and images were captured.

### Mitochondrial staining

Cells were grown in 24-well plates covered with cell crawlers. Cells were treated according to the experimental grouping, and the medium was aspirated. Subsequently, cells were incubated with 100 nM prewarmed MitoTracker Red CMXRos staining solution for 30 min at 37 °C. Cells were then washed thrice with PBS and fixed with a freshly prepared and prewarmed buffer containing 4% paraformaldehyde at 37 °C for 15 min. After fixation, cells were washed thrice with PBS. Glass slides were prepared, and 5 μl of antifluorescence attenuation sealer containing DAPI was used to seal the slides. The slides were observed and imaged using a confocal laser scanning microscope (Nikon, Japan).

### Hematoxylin and eosin and Verhoeff–van Gieson staining

Histopathological analysis of the abdominal aorta of each group was performed using hematoxylin and eosin (H&E) and Verhoeff–van Gieson (EVG) staining. Slices of 6 μm in thickness were prepared. Then, xylene and ethanol were used to dewax the slices, water was drained, and the slices were set aside. H&E and EVG staining was performed using H&E and EVG staining kits, respectively (Solarbio Science&Technology Co., Ltd, Beijing, China). For H&E staining, the slices were first stained with hematoxylin for 15 min and then with eosin for 5 min. For EVG staining, slices were stained with EVG staining solution for 20 min and then differentiated with a differentiation solution for 10 to 20 s. The slices were treated with 95% ethanol for 4 min and restained with eosin for 1 min. Subsequently, an anhydrous ethanol gradient was used to dehydrate the slices, and xylene was used to make the slices transparent. Finally, the slices were sealed and scanned.

### Masson’s trichrome staining

Slices were dewaxed, stained with hematoxylin for 5 to 10 min, treated with hydrochloric acid–alcohol, and washed in running water. The slices were washed with distilled water for 1 min, subsequently stained with an acidic magenta solution for 5 to 8 min, and washed with distilled water. The slices were stained with 1% phosphomolybdic acid for 1 to 3 min and then stained with aniline blue solution for 5 min. They were then washed with water and dried at 60 °C in an oven. The slices were treated with xylene to make them transparent and then sealed with neutral gum. Finally, microscopic examination, image acquisition, and analysis were performed.

### Statistical analysis

All data are represented as the means ± standard error of the mean (SEM). GraphPad Prism v.8.0 (San Diego, CA, USA) was used for statistical analysis. Normality distribution of the data was evaluated using the Shapiro–Wilk test. To compare normally distributed data between 2 groups, Student’s *t* test was performed, and Welch correction was performed if no equal standard deviation was assumed through the *F* test. For comparisons among 3 or more groups of normally distributed data, analysis of variance (ANOVA) or Welch’s ANOVA was performed assuming equal or unequal variances, and each test was compared with those of the other groups using Tukey’s multiple comparison test. If the data were not normally distributed, the Mann–Whitney *U* test was performed for 2 groups or Kruskal–Wallis test for 2 or more groups, and Dunn’s multiple comparisons were performed. The survival rate of mice was tested using the Kaplan–Meier survival curve, and differences were analyzed using the logarithmic rank (Mantel–Cox) test. All statistical analyses used 2-tailed probability values. *P* < 0.05 was considered significant.

## Data Availability

Data are available on request for corresponding author.
