# Supplementary material for: Lactylation of Mitochondrial Adenosine Triphosphate Synthase Subunit Alpha Regulates Vascular Remodeling and Progression of Aortic Dissection
Source: Research (Wash D C). 2025 Aug 12;8:0799. doi: 10.34133/research.0799 (PMC12342782; doi:10.34133/research.0799)
Supplement: Supplementary 1 — Figs. S1 to S7 [file research.0799.f1.docx]

SUPPLEMENTARY MATERIALS

**Figure S1. Reducing lactate production within VSMC can inhibit lactylation**

**
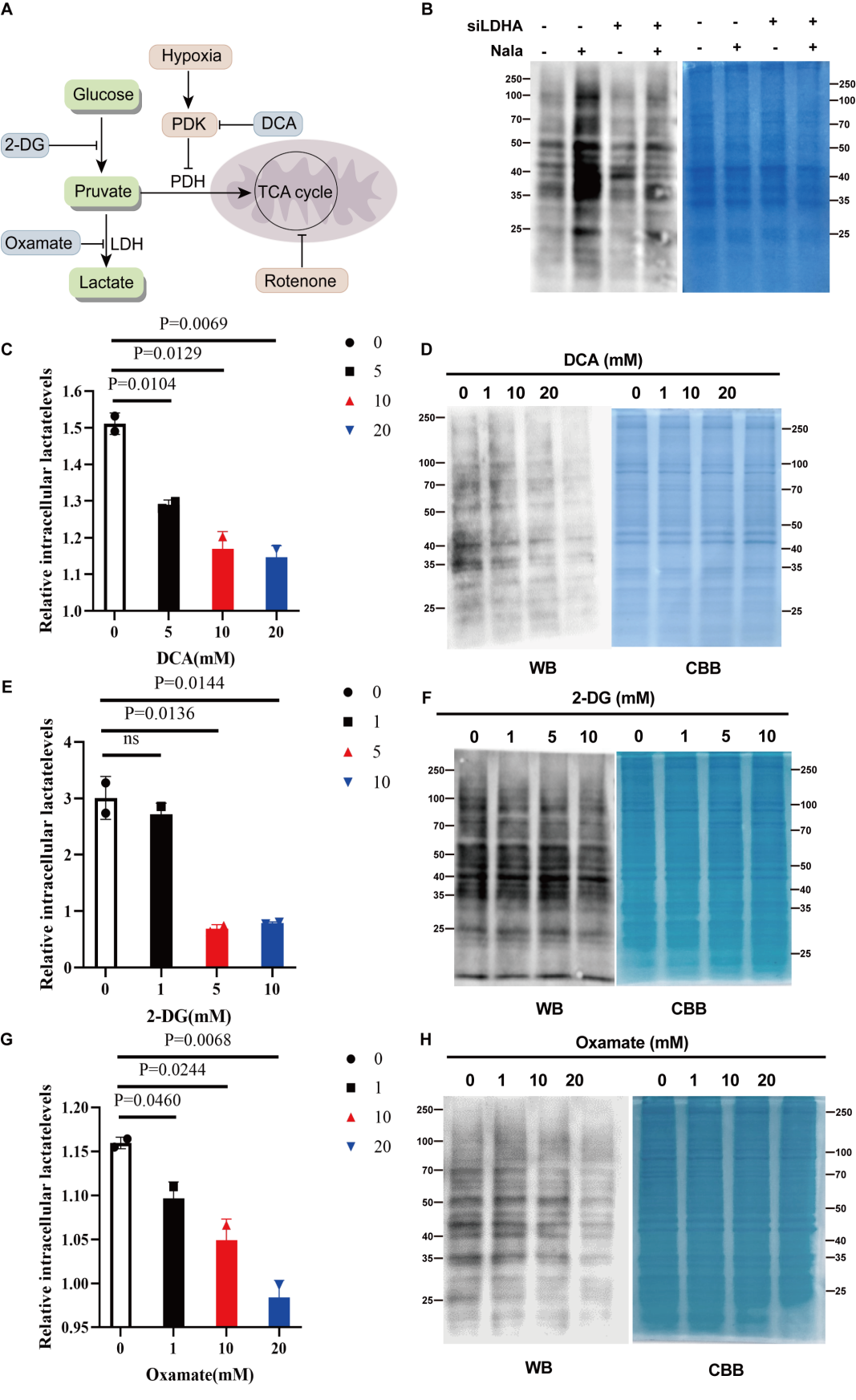
**

A, Regulation of glycolysis and lactate production by diverse metabolic modulators. PDK, pyruvate dehydrogenase kinase; 2-DG, 2-Deoxy-D-glucose; DCA, Dichloroacetate; TCA cycle, tricarboxylic acid cycle. B, Pan-lactylation immunoblots after knockdown of LDHA and application of Nala within VSMC. Nala, Sodium lactate. C and D, Level of intracellular lactate (C) and pan-lactylation immunoblots (D) after treatment with different concentrations of DCA (0, 1, 10, 20 mM) for 24h. E and F, Level of intracellular lactate (E) and pan-lactylation immunoblots (F) after treatment with different concentrations of 2-DG (0, 1, 5, 10 mM) for 24h (n = 3). G and H, Level of intracellular lactate (G) and pan-lactylation immunoblots (H) after treatment with different concentrations of oxamate (0, 1, 10, 20 mM) for 24h (n = 3). Data are means ± SEM from 3 independent experiments, and were analysed by Kruskal–Wallis test with Dunn post-hoc test (C, E and G), *P＜0.05, **P＜0.01, ***P＜0.001.

**Figure S2. Elevation of lactate increases lactylation within VSMC**

**
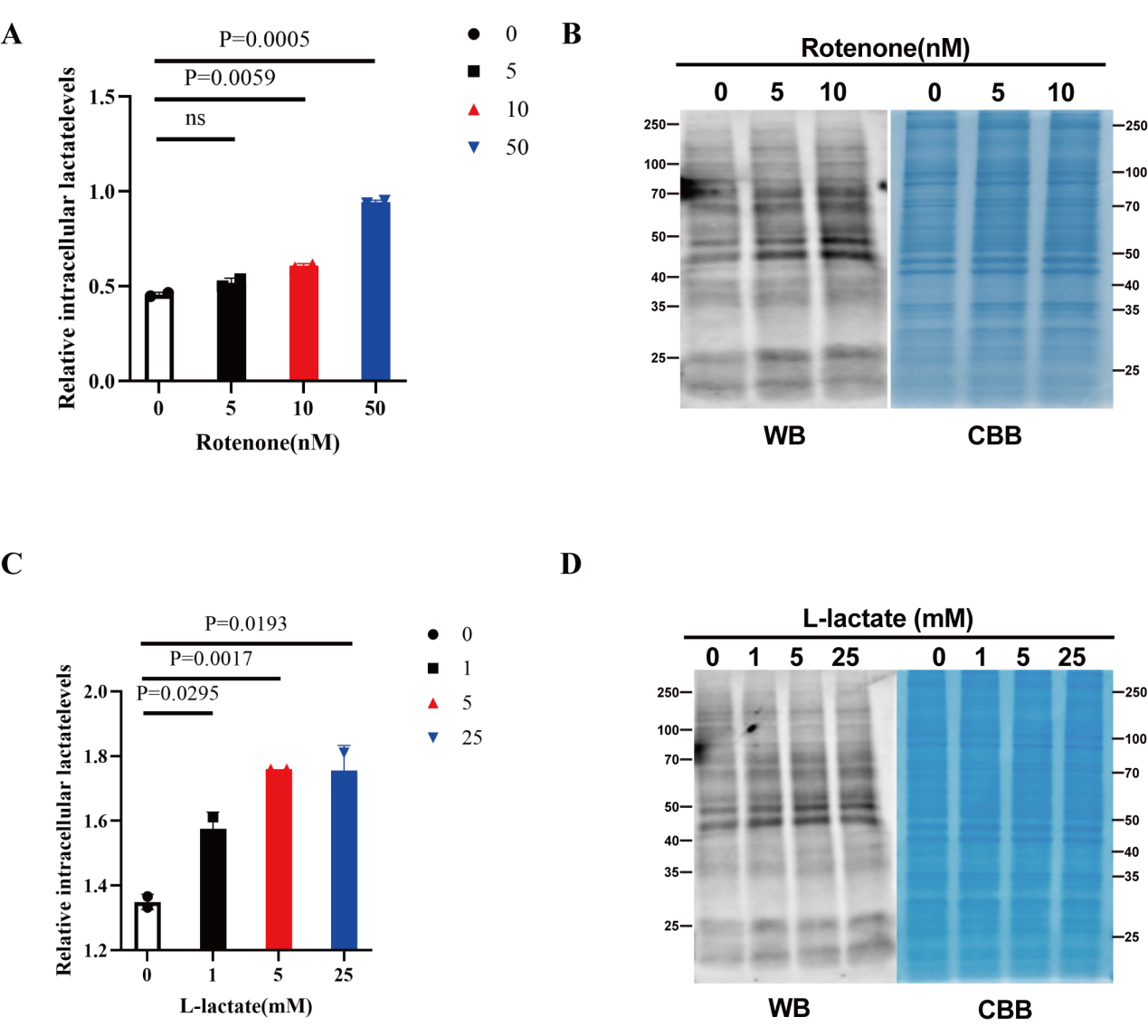
**

A and B, Level of intracellular lactate (A) and pan-lactylation immunoblots (B) after treatment with different concentrations of rotenone (0, 5, 10 nM) for 24h (n = 3). C and D, Level of intracellular lactate (C) and pan-lactylation immunoblots (D) after treatment with different concentrations of lactate (0, 1, 5, 25 mM) for 24h (n = 3). Data are means ± SEM from 3 independent experiments, and were analysed by Kruskal–Wallis test with Dunn post-hoc test (A and C), *P＜0.05, **P＜0.01, ***P＜0.001.

**Figure S3. Lactylation analysis**

**
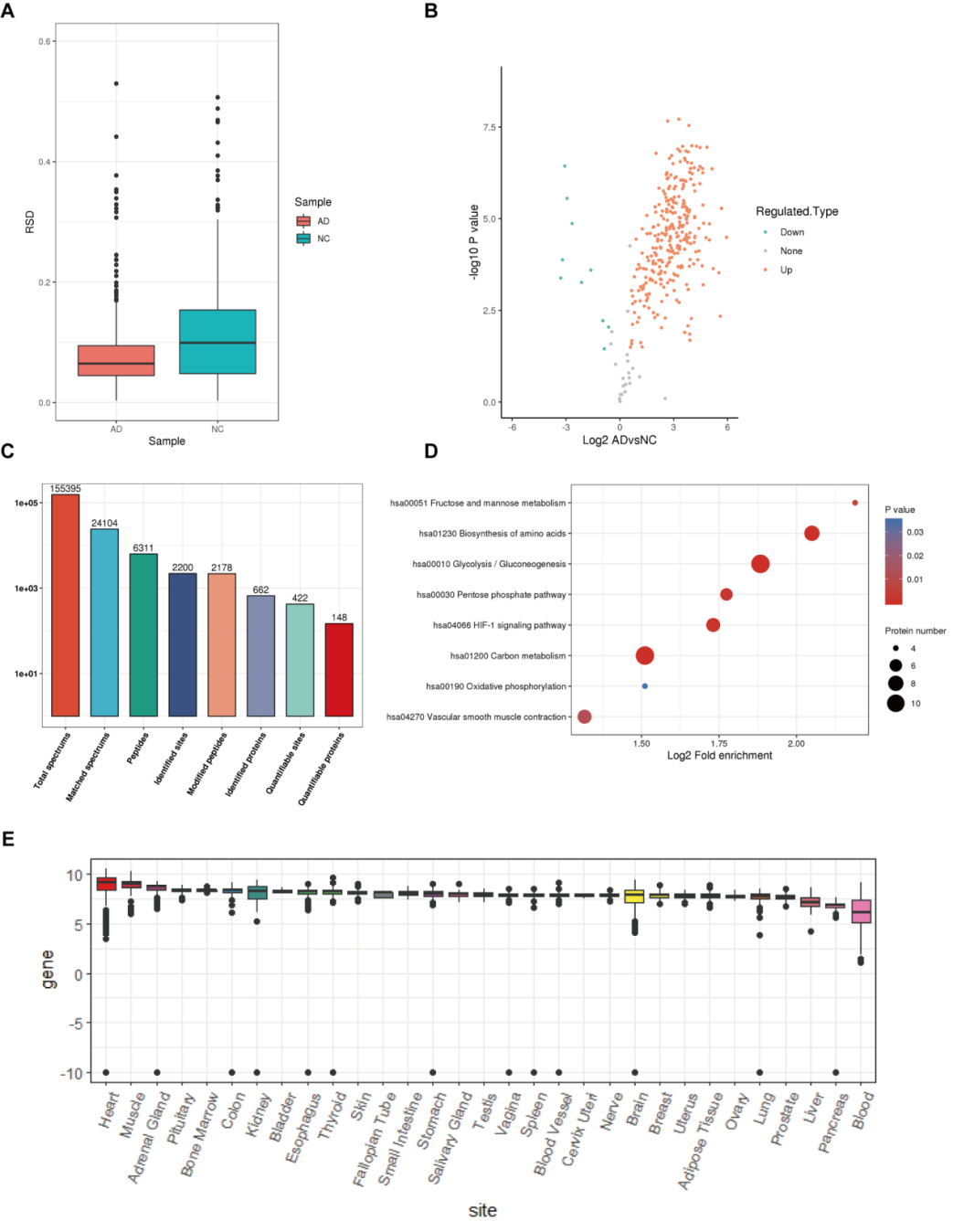
**

A, Box plot of relative standard deviation (RSD) of protein quantification values between repeated samples (n = 3). B, Volcano map of differential modification site. C, Proteins and sites identified by proteomics for upregulation of lactylation in AD. D, KEGG pathway of differentially modified proteins. E, The expression level of ATP5F1A in different tissues.

**Figure S4. Construction of mutant plasmid**

**
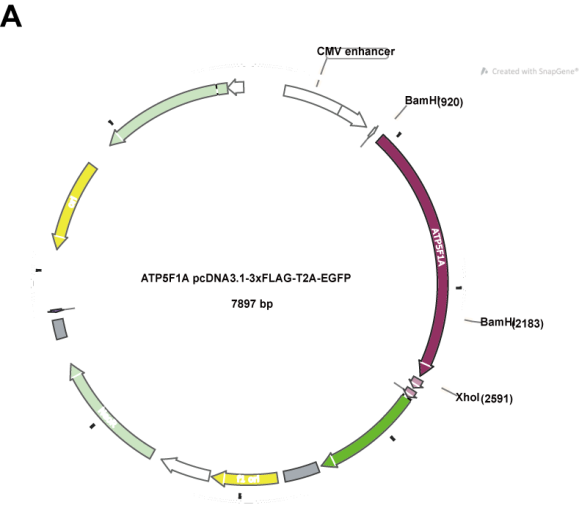
**

A, Construction map of ATP5F1A wild type plasmid.

**Figure S5. K531 lactylation of ATP5F1A regulates VSMC proliferation, migration and phenotypic switching**


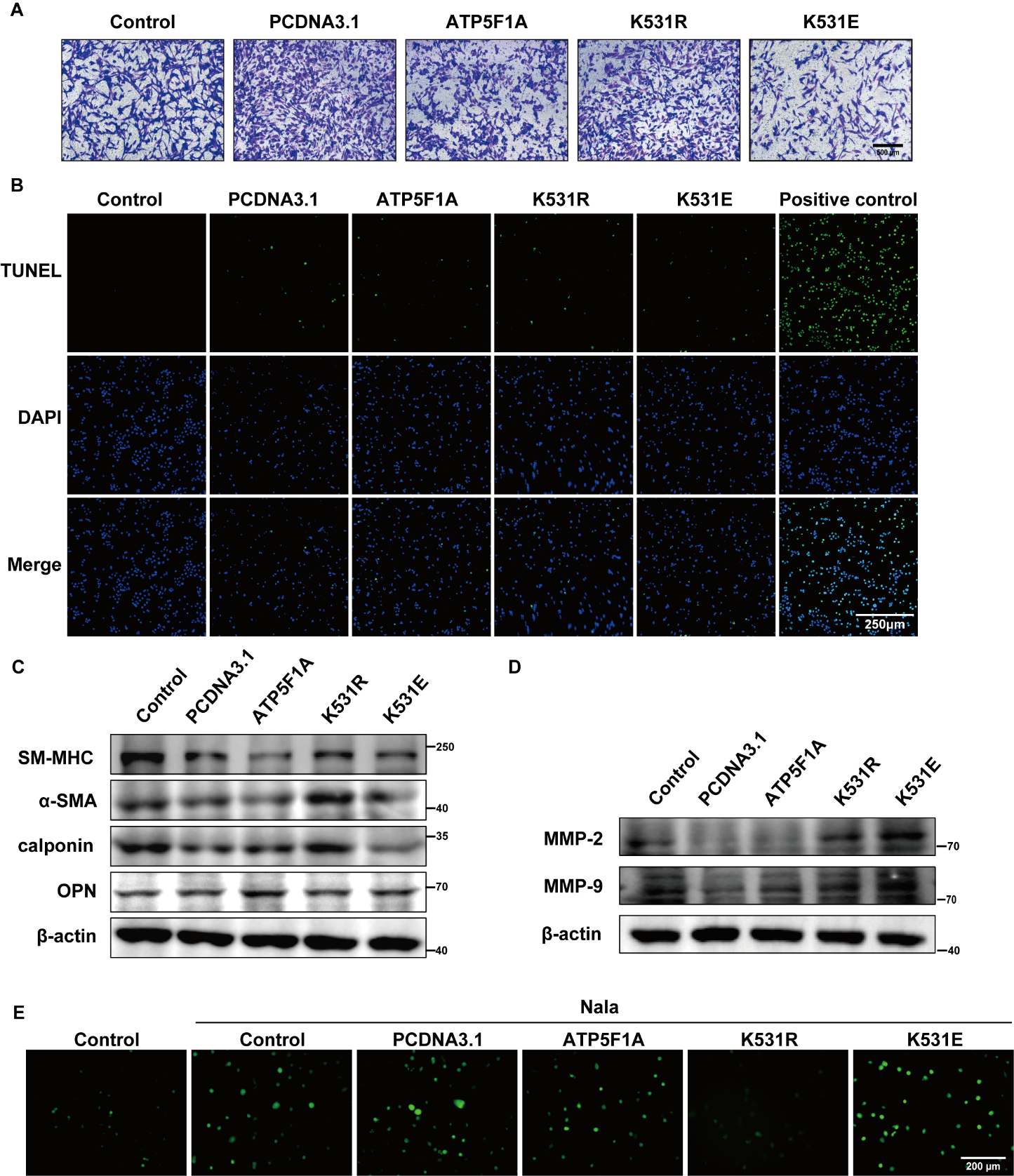


A, Cell migration of HAVSMCs following the ATP5F1A K531 site mutation was detected using crystal violet dye solution (Scale bar = 500 μm). B, Detection of apoptosis in HAVSMCs after the ATP5F1A K531 site mutation with Nala treatment using TUNEL staining (Scale bar = 500 μm). C and D Western Blots was conducted to measure the protein expression level of phenotypic switch  markers (C) and MMP2/9 (D) after ATP5F1A K531 site mutation with Nala treatment. E, Detection of cellular reactive oxygen species production with Nala treatment by DCFH-DA fluorescent staining (Scale bar = 200 μm). Nala, Sodium lactate.

**Figure S6. K531 lactylation of ATP5F1A affects ATP5F1A protein expression**

**
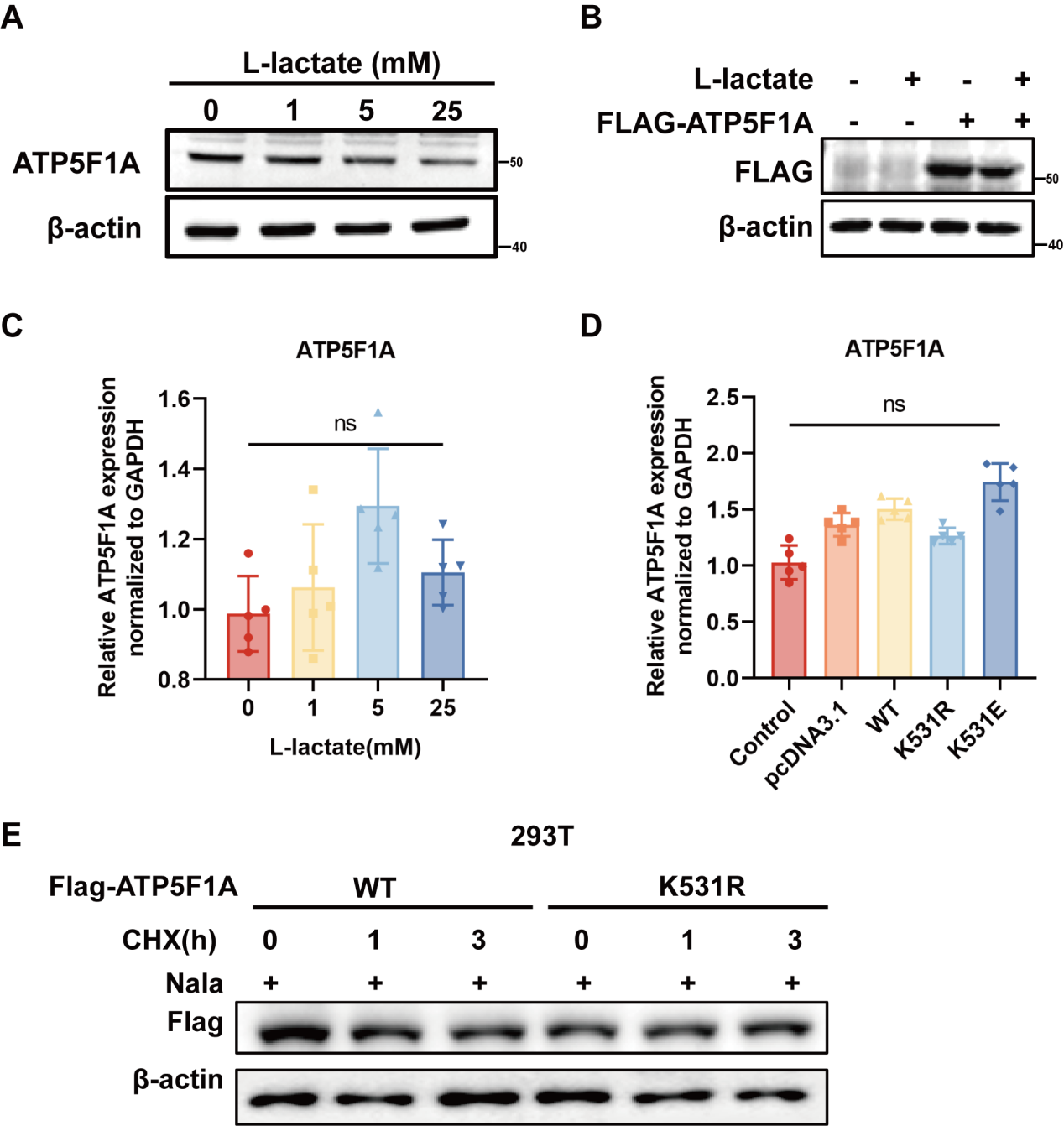
**

A, Detection of ATP5F1A expression levels in VSMC after treatment with different concentrations of lactate (0, 1, 5, 25 mM) for 24 hours using Western Blots. B, Flag-ATP5F1A transfection of HAVSMCs was performed to achieve ATP5F1A overexpression, followed by treatment with lactate. Western Blots were used to detect Flag expression level. C, Relative ATP5F1A mRNA expression was detected using qPCR after treating HAVSMCs with various concentrations of lactate (0, 1, 5, 25 mM), (n = 3). D, Relative ATP5F1A mRNA expression was detected using qPCR after ATP5F1A K531 site mutation (n = 3). E, Wild type and K531R mutant of ATP5F1A transfected with 293T cells were used to detect the expression changes of exogenous ATP5F1A after CHX treatment at different times. Data are means ± SEM from 3 independent experiments, and were analysed by Kruskal–Wallis test with Dunn post-hoc test (C and D), *P＜0.05, **P＜0.01, ***P＜0.001.

**Figure S7. Validation of ATP5F1A binding protein**


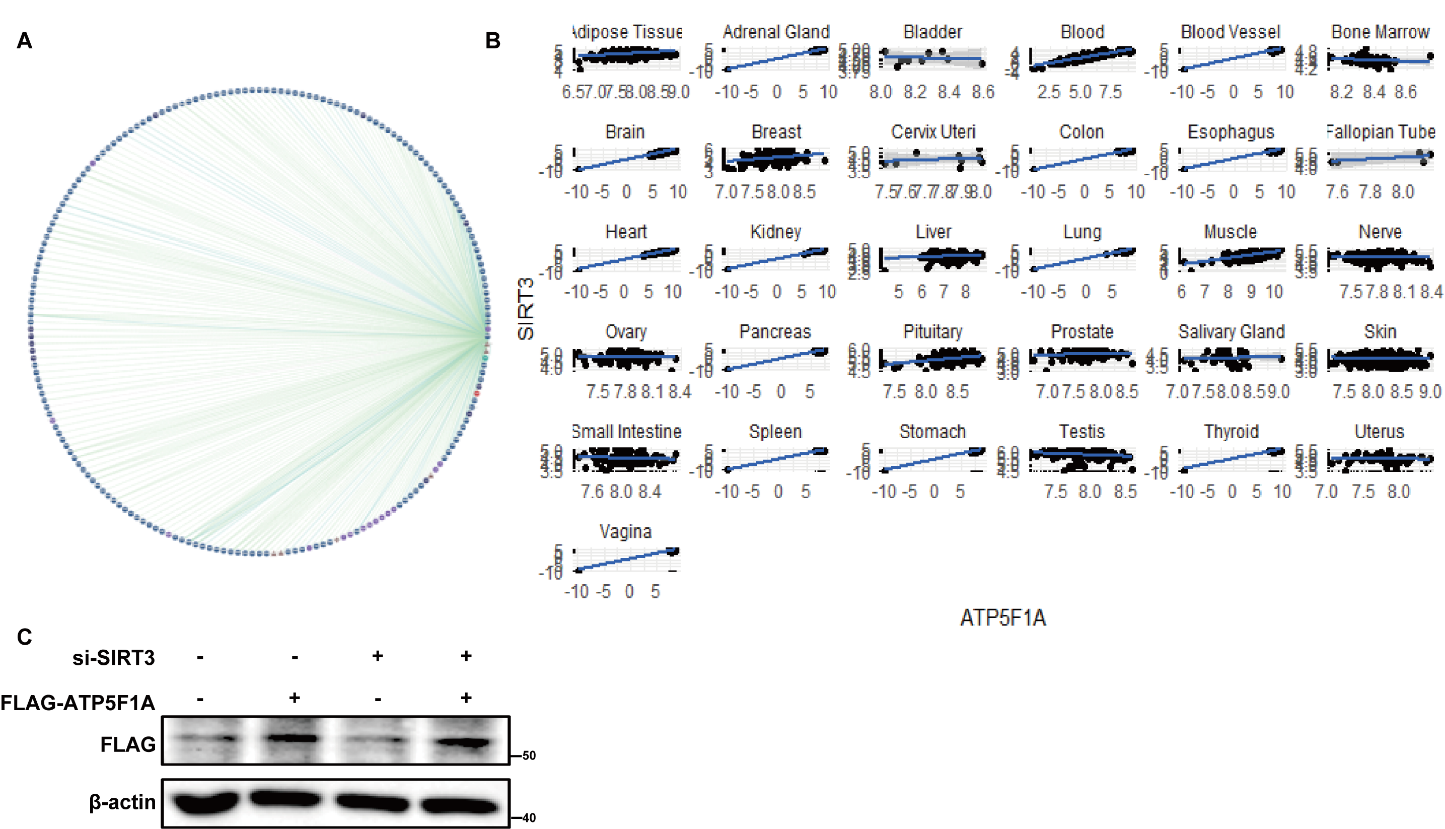


A, ATP5F1A binding protein predicted by INTACT website. B, Correlation analysis of ATP5F1 and Sirt3 in different tissues. C, Western Blots were conducted to measure the expression level of ATP5F1A after knocking down Sirt3.
